# Discovery of Ruthenium(II) Metallocompound and Olaparib Synergy for Cancer Combination Therapy

**DOI:** 10.1021/acs.jmedchem.3c00322

**Published:** 2023-05-15

**Authors:** Nur Aininie Yusoh, Paul R. Tiley, Steffan D. James, Siti Norain Harun, Jim A. Thomas, Norazalina Saad, Ling-Wei Hii, Suet Lin Chia, Martin R. Gill, Haslina Ahmad

**Affiliations:** † UPM-MAKNA Cancer Research Laboratory, Institute of Bioscience, Universiti Putra Malaysia, UPM, 43400 Serdang, Selangor, Malaysia; ‡ Department of Chemistry, Faculty of Science and Engineering, Swansea University, Swansea SA2 8PP, U.K.; § Department of Chemistry, Faculty of Science, Universiti Putra Malaysia, UPM, 43400 Serdang, Selangor, Malaysia; ∥ Department of Chemistry, University of Sheffield, Sheffield S3 7HF, U.K.; ⊥ Center for Cancer and Stem Cell Research, Development and Innovation (IRDI), Institute for Research, International Medical University, Kuala Lumpur 57000, Malaysia; # Department of Microbiology, Faculty of Biotechnology and Biomolecular Science, Universiti Putra Malaysia, UPM, 43400 Serdang, Selangor, Malaysia

## Abstract

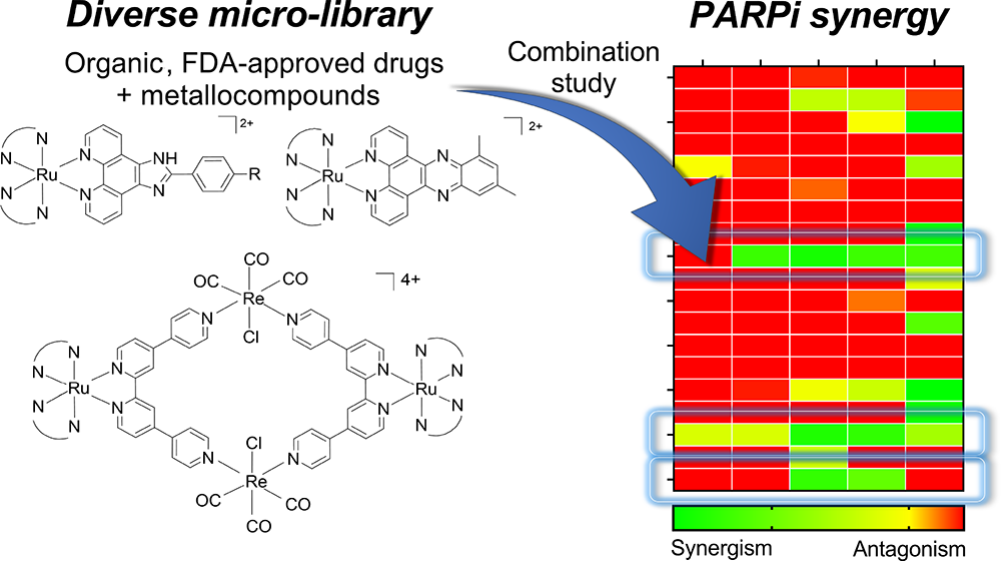

Synergistic drug combinations can extend the use of poly(ADP-ribose) polymerase inhibitors (PARPi) such as Olaparib to BRCA-proficient tumors and overcome acquired or de novo drug resistance. To identify new synergistic combinations for PARPi, we screened a “micro-library” comprising a mix of commercially available drugs and DNA-binding ruthenium(II) polypyridyl complexes (RPCs) for Olaparib synergy in BRCA-proficient triple-negative breast cancer cells. This identified three hits: the natural product Curcumin and two ruthenium(II)-rhenium(I) polypyridyl metallomacrocycles. All combinations identified were effective in BRCA-proficient breast cancer cells, including an Olaparib-resistant cell line, and spheroid models. Mechanistic studies indicated that synergy was achieved via DNA-damage enhancement and resultant apoptosis. Combinations showed low cytotoxicity toward non-malignant breast epithelial cells and low acute and developmental toxicity in zebrafish embryos. This work identifies RPC metallomacrocycles as a novel class of agents for cancer combination therapy and provides a proof of concept for the inclusion of metallocompounds within drug synergy screens.

## Introduction

PARP inhibitors (PARPi) such as Olaparib are now under clinical investigation in both single-agent and combination treatment regimens,^1^ and they achieve their effects by preventing the repair of DNA single-strand breaks (SSBs) or stalled replication forks, generating cytotoxic DNA double-strand breaks (DSBs) that trigger cell death by apoptosis.^2^ Through synthetic lethality, cancers with deficient BRCA pathways are hypersensitive to PARP inhibition^3^ and, as a consequence, PARP inhibitors have been employed as treatments for BRCA-deficient breast cancers to great effect. In these circumstances, they induce a high therapeutic response with low side effects compared to traditional cytotoxic chemotherapy.^4^ However, use of PARPi as single agents is restricted by the fact that BRCA-deficient cancers account for a relatively small subset of cancers compared to the BRCA wild-type^5^ and the heterogenous nature of cancers means that resistance after early use is common.^6^ This is particularly true for triple-negative breast cancer (TNBC), an aggressive form of the disease with a disproportionately high rate of mortality.^7^

Combination therapy has also proven to be a highly successful cancer strategy; synergistic drug combinations offer improved cancer specificity and reduced side effects compared to single-agent treatment and can also combat the challenge of drug resistance.^8^ As PARP inhibitors rely upon synthetic lethality to exert their cytotoxic effects, a combination of PARPi alongside conventional DNA-damaging chemotherapy such as cisplatin or gemcitabine or ionizing radiation has shown encouraging results in clinical trials.^1,9−11^ Inspired by these efforts, synergistic drug combinations for PARPi or agents that induce “chemical BRCAness” have the potential to expand PARPi clinical usage to include BRCA-proficient cancers.^12^ The most common methodology employed to develop new drug combinations for PARPi is judicious selection of complimentary small molecules based on underlying molecular biology;^13−16^ however, screening chemical libraries also holds the potential to uncover new chemical or biological approaches to achieve drug synergy.^17,18^ Despite this, chemical screens for PARPi synergy are rare, yet the potential for this may be demonstrated by Liu et al. who utilized this to great effect to identify BET, SRC, and BCL2 inhibitors as new combinatorial therapeutics able to overcome PARPi resistance.^19^ This approach has the advantage that chemical diversity can be introduced deliberately, thereby promoting serendipitous discovery and the isolation of new scaffolds for chemically induced synthetic lethality.^20,21^

One route to expanded chemical diversity is presented by organometallic or coordination chemistry as metallocompounds can provide molecular geometries, shapes, and reactivities inaccessible to pure organics.^22^ Numerous metallocompounds have been examined for their anticancer properties, where modulated chemical stability and metal- and/or ligand-based reactivities are cited as advantageous properties.^23^ The most successful have been the platinum drugs, including octahedral Pt(IV) pro-drugs,^24^ while, more recently, substitutionally inert ruthenium(II) polypyridyl complexes (RPCs) have become the subject of increasing interest as potential successors to these platinum systems.^25−27^ The potential of RPCs within this area can be illustrated by work describing RPCs that intercalate between base pairs of DNA, “metallo-intercalators”, that are able to interfere with DNA replication or transcription with distinct mechanisms of action compared to existing DNA targeting agents such as cisplatin.^28−30^ Specific examples include the organometallic complex [Ru(bpy)(phpy)(dppz)]^+^ (bpy = 2,2′bipyridine; phpy = 2-phenylpyridine; dppz = dipyrido[3,2-*a*:2′,3′-*c*]phenazine), which disrupts the transcription factor NF-κB binding to DNA, resulting in low-micromolar half inhibitory IC_50_ concentrations in numerous cancer cell lines^28^ and [Ru(phen)_2_(tpphz)]^2+^ (phen = 1,10-phenanthroline; tpphz = tetrapyridophenazine), which induces replication fork collapse and inhibits esophageal cancer cell proliferation through a combination of S-phase cell-cycle arrest and mitotic arrest.^29^ Notably, both complexes exhibit cytotoxicity comparable to—or greater than—cisplatin but with improved cancer selectivity.

We have described one such RPC, [Ru(dppz)_2_(PIP)]^2+^ (PIP = 2-(phenyl)-imidazo[4,5-*f*][1,10]phenanthroline), “Ru-PIP”, which achieves preferential cancer cell proliferation inhibition by replication fork stalling and corresponding G1/S phase cell-cycle arrest.^31^ Remarkably, sub-cytotoxic concentrations of Ru-PIP render TNBC and lung cancer cells hypersensitive to Olaparib, with a >300-fold increase in Olaparib potency achieved as a result of complementary mechanisms of action,^32,33^ one of the greatest nongenetic PARPi enhancement effects described to date. In this study, we continue these efforts to identify and characterize new synergistic combinations for PARPi in BRCA-proficient TNBC cells. By employing a proof-of-concept “micro-library” comprising a mix of DNA-binding RPCs and commercially available DNA-damaging drugs, a secondary aim was to explore the use of metallocompounds alongside organics for additional chemical diversity in a drug synergy screen. We characterized the mechanism of synergy for newly discovered synergistic combinations in detail and verified the activity in an Olaparib-resistant cell line and tumor spheroids before finally assessing their *in vivo* toxicity in a zebrafish embryo model.

## Results

### Design of a Mixed Metallocompound/Organic DNA-Targeting “Micro-library”

A “micro-library” of 19 compounds was assembled, which was composed of nine commercially available anti-cancer drugs or drug candidates and 10 DNA-binding RPCs. Compounds selected were DNA-damaging chemotherapeutics cisplatin, gemcitabine hydrochloride, and Fluorouracil (5FU), oestrogen antagonist Tamoxifen citrate, PARPi NU1025, ATR (ataxia telangiectasia and Rad3-related protein) inhibitors Berzosertib and Ceralasertib, and natural products Quercetin and Curcumin (Table S1 in the Supporting Information).

**Figure 1:**
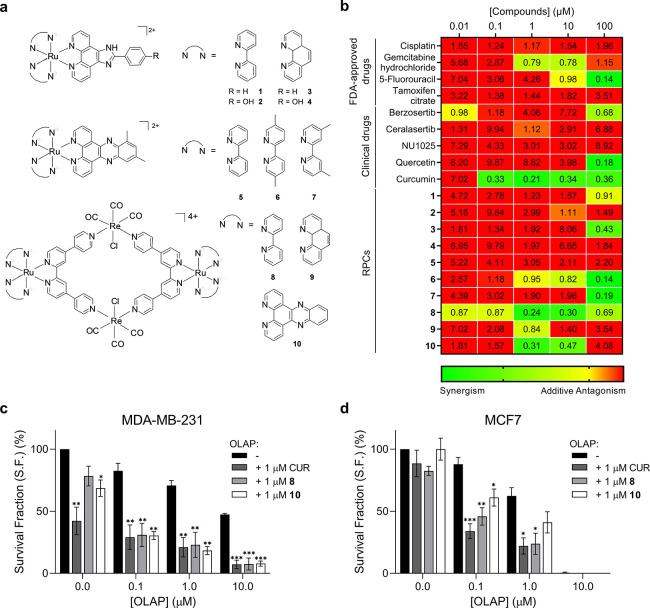
(a) Structures of RPCs employed within this study. Compounds were used as a mixture of enantiomers. (b) Combination indices (CIs) for micro-library compound/Olaparib combinations in MDA-MB-231 cells. Cell viability without or with Olaparib (10 μM) determined after 72 h treatment by the MTT assay (mean of at least two independent experiments). CI values were calculated using CompuSyn software, and a heat map was generated as described in the Experimental Section. Clonogenic survival assays of (c) MDA-MB-231 or (d) MCF7 cells treated with single-agent Olaparib (0.1, 1, or 10 μM) or in combination with low-dose (1 μM) Curcumin (CUR), **8**, or **10** (72 h treatment time). Data expressed as mean ± SEM of three independent experiments. *P < 0.05, **P < 0.01, and ***P < 0.001 compared to the Olaparib single agent-treated group by ANOVA.

RPCs utilized three distinct scaffolds: [Ru(N^N)_2_(PIP)]^2+^ (**1**–**4**), [Ru(N^N)_2_(dmdppz)]^2+^ (dmdppz = 10,12-dimethyl-dipyrido[3,2-*a*:2′,3′-*c*]phenazine) (**5**–**7**), or Ru(II)-Re(I) qtpy metallomacrocycles (qtpy = 2,2′:4,4″:4′,4″′ quaterpyridyl) (**8**–**10**) (Figure 1a). RPCs were synthesized by established pathways^34−38^ and characterized by ^1^H NMR, mass spectrometry, and elemental analysis. Results for previously reported compounds (**1**–**4** and **8**–**10**) were in agreement with published data.^34,35^ Novel compounds **5**–**7** were characterized by ^1^H and ^13^C NMR, high-resolution mass spectrometry, elemental analysis, HPLC, and FT-IR (Figures S1–S8). All RPCs bind DNA by reversible binding with medium to high affinity, and luminescence titrations indicated that the three novel [Ru(N^N)_2_(dmdppz)]^2+^ complexes demonstrated the highest DNA binding affinities (binding affinities (*K*_b_) = 8.7 × 10^6^, 9.2 × 10^6^, and 5.7 × 10^6^ M^–1^ for **5**, **6**, and **7** respectively, Figure S9 and Table S2). In terms of DNA binding mode, compounds **1**–**7** and **10** contain established intercalating ligands PIP, H-PIP (2-(4-hydroxyphenyl)imidazo[4,5-*f*][1,10]phenanthroline), dppz, or dmdppz,^39,40^ while **8** and **9** bind DNA via non-intercalative mechanisms.^41,42^ As a result, all compounds either bind DNA in cell-free conditions with medium to high affinity, induce DNA damage by targeting DNA in cells or inhibiting DNA repair, or have been reported to induce cellular DNA damage in mechanistic studies (Tables S1 and S2).

### Olaparib Co-treatment Identifies New Synergistic Combinations

To identify synergistic combinations with Olaparib, BRCA-proficient MDA-MB-231 TNBC cells were treated with a concentration gradient of each micro-library compound in the absence or presence of low-dose, non-cytotoxic (10 μM) Olaparib (Figure S10). Resultant cell viabilities at 72 h were determined by an MTT assay, and these data were used to derive half inhibitory IC_50_ concentrations (Figure S11 and Table 1).

**Table 1:** Half Maximal Inhibitory Concentration (IC_50_) Values of Each Compound Alone or in Combination with Olaparib (10 μM)^a^

	–OLAP	+OLAP	
compound	IC_50_ (μM)	IC_50_ (μM)	fold shift
cisplatin	35.3 ± 5.5	26.1 ± 11.1	1.4
gemcitabine hydrochloride	1.6 ± 0.8	0.6 ± 0.1	2.7
Fluorouracil	12.1 ± 3.0	4.2 ± 0.3	2.9
Tamoxifen citrate	20.6 ± 1.3	19.9 ± 4.7	1.0
Berzosertib	0.46 ± 0.01	0.27 ± 0.06	1.7
Ceralasertib	4.4 ± 0.4	5.0 ± 1.5	0.9
NU1025	>100	>100	ND
Quercetin	>100	7.1 ± 5.7	>14.1
Curcumin	25.1 ± 2.4	0.09 ± 0.01	278.9
**1**	>100	75.5 ± 12.2	>1.3
**2**	>100	>100	ND
**3**	29.2 ± 3.7	8.0 ± 3.3	3.7
**4**	11.6 ± 3.2	1.7 ± 0.9	6.8
**5**	75.1 ± 12.2	>100	<0.8
**6**	87.4 ± 2.3	17.4 ± 5.3	5.0
**7**	34.3 ± 0.5	15.2 ± 2.5	2.3
**8**	80.9 ± 8.2	0.6 ± 0.3	134.8
**9**	>100	57.4 ± 10.2	>1.7
**10**	26.3 ± 5.1	1.0 ± 0.2	26.3

a MDA-MB-231 72 h. IC_50_ values determined by the MTT assay. Data expressed as mean ± SD of at least two independent experiments. Fold shift = IC_50_ (−OLAP)/IC_50_ (+OLAP). ND = not determined.

Examining the effects of each compound as a single agent, the most cytotoxic molecule tested was the ATR inhibitor Berzosertib (IC_50_ = 460 ± 10 nM) and **4** was the most cytotoxic RPC (IC_50_ = 11.6 ± 3.2 μM), despite having the lowest DNA binding affinity (*K*_b_ = 6.9 × 10^4^ M^–1^, Table S2). To assess Olaparib synergy, Chou and Talalay combination indices (CIs)^43^ were calculated for each concentration tested and CI scores of less than 0.9 were considered synergistic (Figure 1b). This analysis identified two clear hits for synergy with Olaparib: Curcumin and **8** were both synergistic over most of the tested concentrations, while **10** was scored as a moderate hit, with 40% of the concentrations tested exhibiting synergy with Olaparib. The associated IC_50_ values illustrate the magnitude of synergy demonstrated by these three compounds. In each case, a large increase in potency is generated on inclusion of Olaparib (a 279-, 135-, and 26-fold decrease in IC_50_ values for Curcumin, **8**, and **10** respectively, Table 1).

To validate the synergistic pairs isolated by this screen, clonogenic survival assays on the three hits were performed in the presence and absence of Olaparib. These demonstrated that MDA-MB-231 cells treated with a low (1 μM) dose of Curcumin, **8**, or **10** display substantially enhanced cell sensitivity to Olaparib (a >1000, >143, and >200 increase in Olaparib potency with the addition of Curcumin, **8**, and **10**, respectively; Figure 1c and Table S3). As **8** and **10** possess a low impact on colony formation at this concentration (survival fractions (S.F.) > 70%), this confirms synergy. Also, for their effect on MDA-MB-231 cells, combinations were also found to be synergistic in BRCA-proficient MCF7 human breast cancer cells in both MTT and clonogenic survival assays (Figure 1d, Figures S12 and S13, Table 2, and Table S3).

**Table 2:** IC_50_ Values of Curcumin, **8**, or **10** Alone or in Combination with 10 μM Olaparib Following 72 h Treatment in MCF10A, MDA-MB-231, or MCF7 Cells and Their Respective Selectivity Indices (SI)^a^

		IC_50_ (μM)			SI	
compound(s)		MCF10A	MDA-MB-231	MCF7	MDA-MB-231	MCF7
untreated	+OLAP	>100	80.9 ± 15.0	69.6 ± 6.3	>1.2	>1.4
Curcumin	–OLAP	37.4 ± 6.0	25.1 ± 2.4	22.5 ± 4.1	1.5	1.7
	+OLAP	34.7 ± 8.9	0.09 ± 0.01	0.02 ± 0.01	385.6	1735
**8**	–OLAP	60.0 ± 6.4	80.9 ± 8.2	38.0 ± 15.3	0.7	1.6
	+OLAP	31.4 ± 5.6	0.6 ± 0.3	0.01 ± 0.01	52.3	3140
**10**	–OLAP	>100	26.3 ± 5.1	>100	>3.8	ND
	+OLAP	>100	1.0 ± 0.2	0.02 ± 0.01	>100	>5000

a IC_50_ values determined by the MTT assay. Data expressed as mean ± SD of at least two independent experiments (*n* = 3). SI = IC_50_ MCF10A/IC_50_ cancer cell line. Results for MDA-MB-231 cells from Table 1 included for comparison. ND = not determined

To examine cancer selectivity, single agents and combinations were tested in MCF10A normal human breast cells. These results show mild or no cancer selectivity for Curcumin, **8**, or **10** as single agents; however, when each compound is combined with low-dose Olaparib substantial (>50-fold) cancer v. non-cancer cell selectivity is then apparent (Table 2 and Figure S12 in the SI).

### Olaparib Drug Synergy Identifies New DNA-Damaging Agents

**Figure 2:**
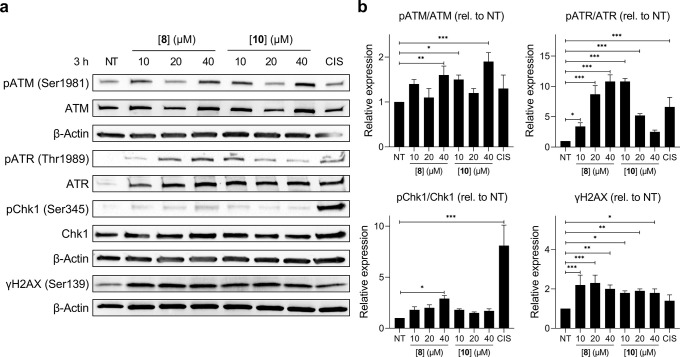
(a) Western blot analysis of DNA damage response activation in MDA-MB-231 cells treated with **8** or **10** for 3 h. Cisplatin (CIS) treatment (50 μM, 3 h) was included for comparative purposes. (b) Quantification of results in (a) by densitometry. NT = untreated. Data expressed as mean ± SD of triplicate experiments. *P < 0.05, **P < 0.01, and ***P < 0.001 by ANOVA.

As PARP enzymes are responsible for mediating DNA damage repair pathways,^44^ the observed synergy of **8** and **10** with the PARPi Olaparib would imply that each molecule induces DNA damage as part of its mechanism of action. Exploring this possibility, Figure 2a,b shows that MDA-MB-231 cells treated with a concentration gradient of **8** or **10** for 3 h increased the levels of several DDR signaling proteins in a statistically significant manner, including activated (phosphorylated) p-ATR (at Thr1989), p-ATM (at Ser1981, where ATM = ataxia-telangiectasia mutated), and γH2AX (H2AX phosphorylated at Ser139) (Figure 2). Early activation of these DDR signaling pathways by **8** and **10** is consistent with each molecule inducing both single-strand break (SSB) and double-strand break (DSB) DNA damage, and these results also indicate that our Olaparib synergy screen has successfully identified new cellular DNA damaging agents.

### Mechanistic Basis for Olaparib Synergy

**Figure 3:**
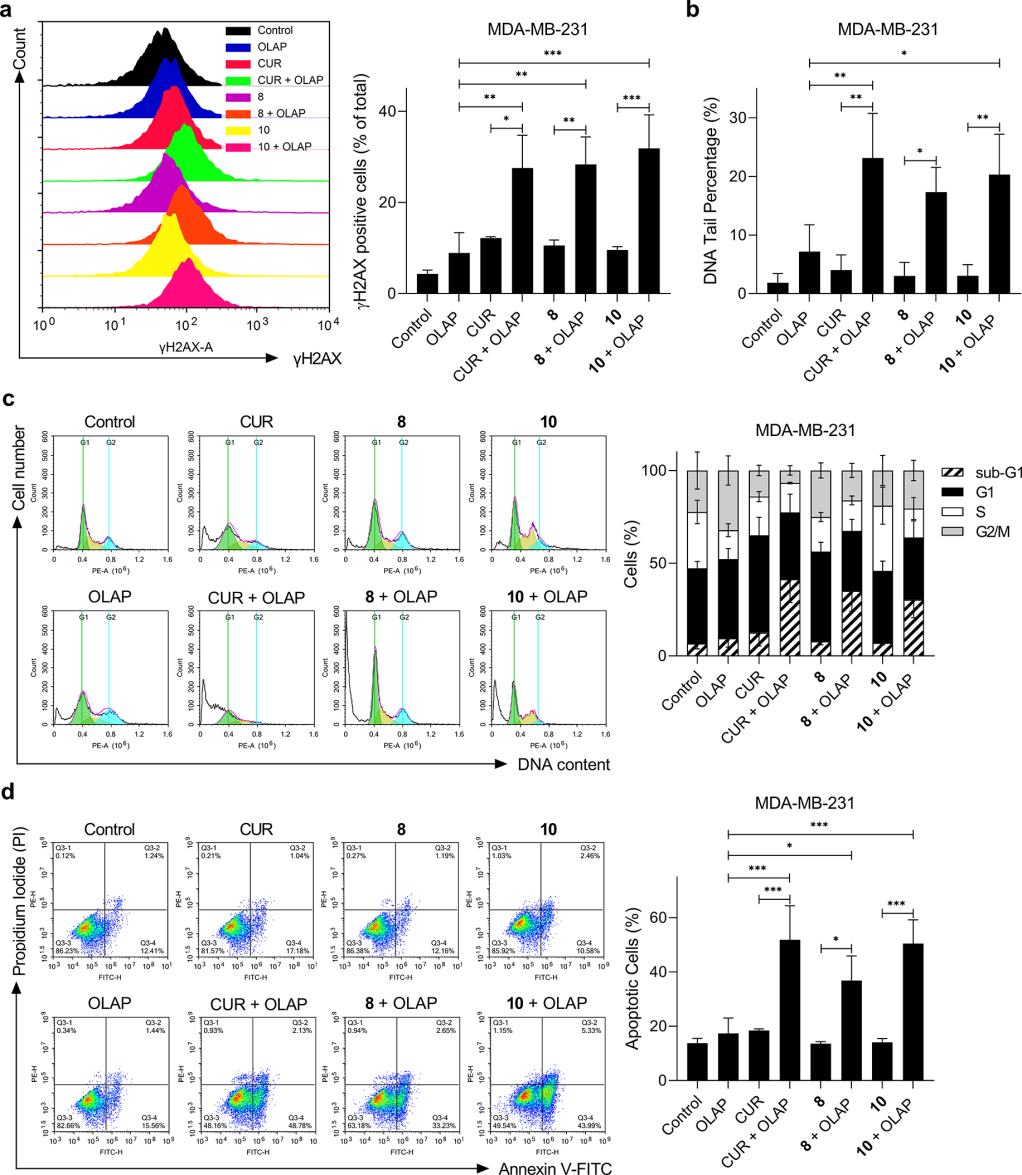
(a) γH2AX levels upon treatment with the stated single agent (1 μM) alone or in combination with Olaparib (10 μM) for 24 h in MDA-MB-231 cells, determined by immunofluorescence and flow cytometry analysis. The percentage of γH2AX-positive cells in each population was determined by gating of histograms derived from single-stained cells. Left, representative histograms, right, quantified data. (b) Quantification of DNA damage by the alkaline comet assay for cells treated as in (a). DNA damage assessed by DNA tail % where at least 100 nucleoids were analyzed per sample. (c) Cell-cycle distribution of MDA-MB-231 cells treated with the stated single agent (1 μM) alone or in combination with Olaparib (10 μM) for 72 h, as determined by PI staining and flow cytometry. Left, representative histograms, right, quantification of the cell-cycle phase. (d) Annexin V-FITC assay of MDA-MB-231 cells treated as in (c). Left, representative scatterplots showing the percentage of cells in each quadrant. Right, quantification of apoptotic cells (Q3-2 and Q3-4 quadrants) for each treatment condition. Data expressed as mean ± SD of three independent experiments. *P < 0.05, **P < 0.01, and ***P < 0.001 by ANOVA.

Examining the mechanism of synergy of **8** and **10** with Olaparib, greater γH2AX levels are apparent in cells treated with the combinations compared to their single-agent equivalents, evidenced by both immunostaining and immunofluorescence (2.3-fold for CUR + OLAP vs CUR; 2.7-fold for **8** + OLAP vs **8**; 3.3-fold for **10** + OLAP vs **10**; *P* < 0.05; Figure 3a and Figure S14). This finding was confirmed by the alkaline comet assay, where significantly greater DNA tail lengths were measured in all three co-treatment conditions compared to as a single agent (5.7-fold for CUR + OLAP vs CUR; 5.7-fold for **8** + OLAP vs **8**; 6.6-fold for **10** + OLAP vs **10**; *P* < 0.05; except *P* > 0.05 for **8** + OLAP vs OLAP; Figure 3b). Examining the impact on cell-cycle distribution, an increase in cells with the sub-G1 content (Figure 3c) accompanied by elevated levels of Annexin V-positive cells (2.8-fold for CUR + OLAP vs CUR; 2.7-fold for **8** + OLAP vs **8**; 3.6-fold for **10** + OLAP vs **10**; *P* < 0.05; Figure 3d) was apparent in all co-treatment conditions, indicative of high levels of apoptosis, while an increase in intracellular reactive oxygen species (ROS) levels in cells co-treated with Olaparib and **10** was apparent (6.8-fold for CUR + OLAP vs CUR; 2.5-fold for **8** + OLAP vs **8**; 2.8-fold for **10** + OLAP vs **10**; *P* < 0.001; except *P* > 0.05 for **8** + OLAP vs single agents; Figure S15a). As in MDA-MB-231 cells, enhanced DNA damage, Annexin-V positive cells, and ROS levels were all observed in MCF7 cells treated with the three synergistic pairs (Figures S15b and S16). These results are consistent with a mechanism of synergy whereby DNA damage induced by **8** or **10** is unrepaired due to PARP inhibition by Olaparib, resulting in the accumulation of substantial DSB damage that triggers cell death by apoptosis.

### Synergistic Combinations Retain Activity in an Olaparib-Resistant Cell Line

**Figure 4:**
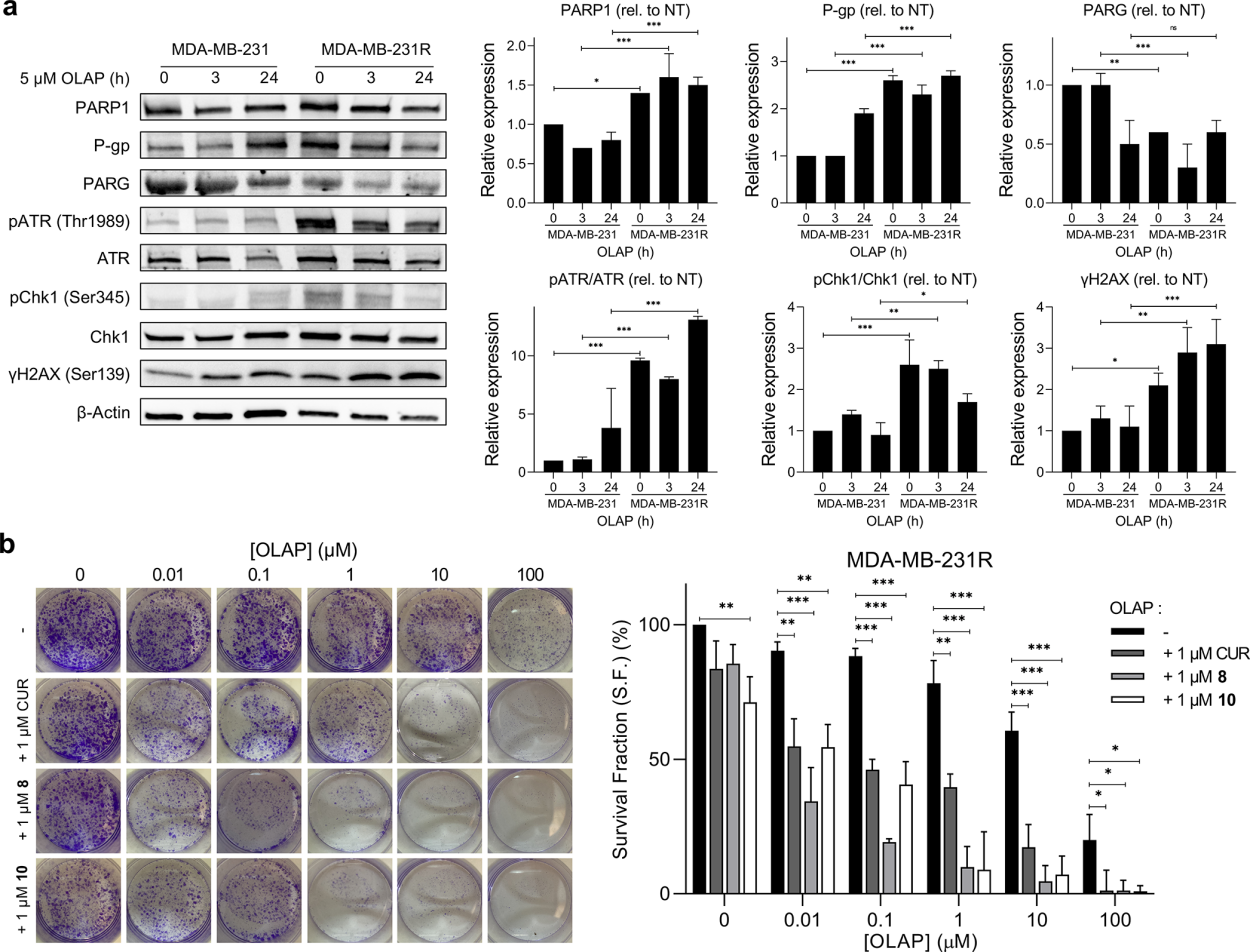
(a) Western blot analysis of selected DNA damage response activation in native MDA-MB-231 cells and derived Olaparib-resistant MDA-MB-231R cells following treatment with 5 μM Olaparib for 0, 3, and 24 h. Data expressed as mean ± SD of triplicate experiments. (b) Clonogenic survival assays of MDA-MB-231R cells treated with single-agent Olaparib (0.01, 1, 10, and 100 μM) or in combination with low-dose (1 μM) Curcumin, **8**, or **10** (72 h treatment time). Data expressed as mean ± SD of three independent experiments. NT = untreated. ns = not significant, *P < 0.05, **P < 0.01, and ***P < 0.001 by ANOVA.

To examine whether our combinations were effective in Olaparib-resistant TNBC, we developed an Olaparib-resistant MDA-MB-231 strain, designated MDA-MB-231R, from the parental MDA-MB-231 cell line by long-term (∼8 months) Olaparib exposure in a similar manner as described by Kim et al.^45^ Olaparib resistance of MDA-MB-231R cells was confirmed by the clonogenic survival assay, where a 28-fold level of resistance to Olaparib treatment for MDA-MB-231R cells compared to parental MDA-MB-231 cells was observed (Figure S17). The acquisition of Olaparib resistance is accompanied by increased basal levels of p-ATR (activated ATR), upregulation of drug efflux pumps such as p-glycoprotein (P-gp), and loss of poly(ADP-ribose) glycohydrolase (PARG) (Figure 4a). This finding is consistent with an up-regulated ATR pathway activation resistance mechanism reported by Kim et al. in ovarian cancer cell lines with acquired PARPi-resistance.^45^ Examining the effectiveness of the newly identified drug combinations in MDA-MB-231R cells, clonogenic survival assays show that the combination of low-dose Curcumin, **8**, or **10** (1 μM) alongside a concentration gradient of Olaparib (0.1–100 μM) retains the ability to inhibit colony formation in a similar manner to the parental MDA-MB-231 cells (Figure 4b).

### Growth Inhibition of Tumor Spheroid Models

**Figure 5:**
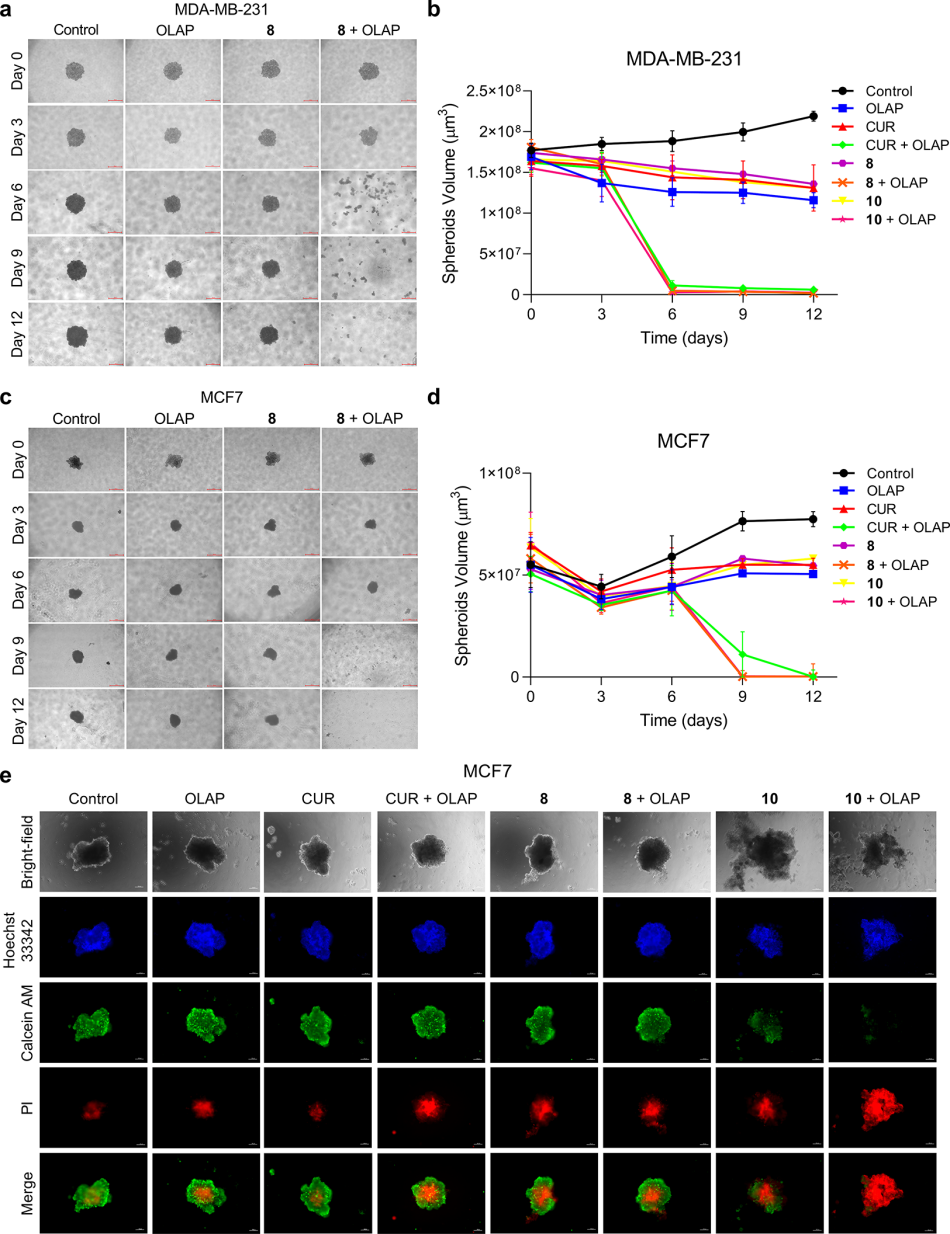
Images of spheroids of (a) MDA-MB-231 or (c) MCF7 breast cancer cells and selected treatment groups. Scale bars = 500 μm. (b, d) Quantification of volume of spheroids treated with the stated single agent (1 μM) alone or in combination with Olaparib (10 μM). Data expressed as mean ± SD of 18 spheroids from three independent experiments. (e) Live/dead staining of MCF7 spheroids following treatments with the stated single agent (1 μM) alone or in combination with Olaparib (10 μM) for 72 h. Live cells indicated by Calcein AM (green), dead cells by propidium iodide (PI, red). DNA staining by Hoechst 33342 (blue) provides an indication of the total cell number. Bright-field image also included. Scale bars = 200 μm.

3D cancer cell spheroids provide an improved model of the internal tumor microenvironment and structure compared to conventional 2-D tissue culture.^46^ We therefore examined the ability of the identified synergistic pairs to inhibit growth of tumor spheroids, comparing results to single-agent treatment conditions. MDA-MB-231 and MCF7 3D tumor spheroids were prepared and incubated with Curcumin, **8**, or **10** in the presence or absence of Olaparib. Spheroids were imaged at 0, 3, 6, 9, and 12 days of incubation, and their sizes were determined. While untreated spheroids showed an increase in size, spheroids treated with combinations of Olaparib and Curcumin, **8**, or **10** showed complete disintegration at day 6 for MDA-MB-231 spheroids (Figure 5a,b) and at day 9 for MCF7 spheroids (Figure 5c,d). In addition to growth studies, live/dead staining was performed on spheroids at 72 h incubation. Visualizing spheroids by fluorescence microscopy, cell death (propidium iodide, PI, positive staining) in the center of spheroids treated with single-agent Curcumin, **8**, or **10** was apparent (Figure 5e), corresponding to a necrotic core. In co-treatment conditions of **10** and Olaparib, almost total cell death was observed, evidenced by the absence of live-cell Calcein staining and abundant PI signal (Figure 5e). As fragmentation appears to be the result of extensive cell death^47^ and did not occur in untreated controls or single-agent treatment conditions, this provides evidence that our synergistic drug combinations are effective in spheroid models.

### Characterization of Acute and Developmental Toxicity in Zebrafish Embryos

**Figure 6:**
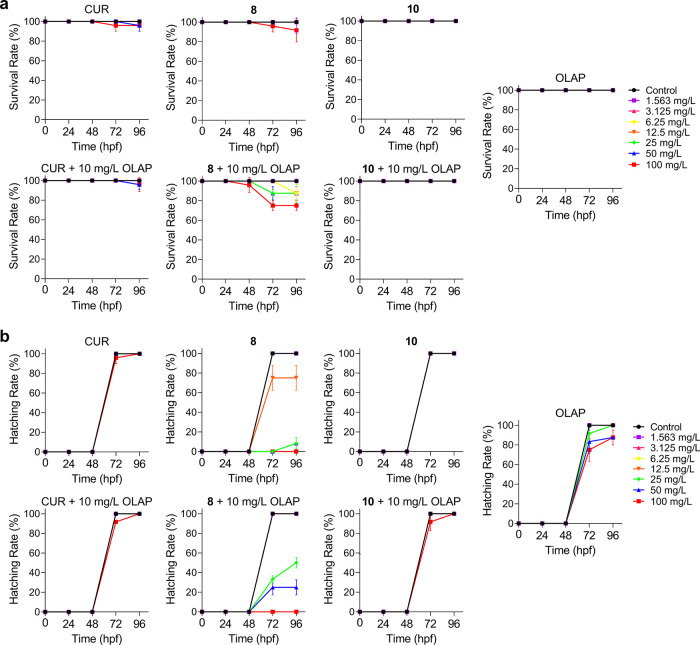
(a) Survival rates and (b) hatching rates of zebrafish embryos upon treatment with the stated single agent alone or in combination with 10 mg/L Olaparib. Data expressed as percentage of survival or hatching of 24 embryos from two independent experiments. hpf = hours post fertilization.

As all synergistic combinations occur with the enhancement of potentially genotoxic DSB damage, an understanding of the toxicity of these compounds and combinations is paramount to their future applications. Accordingly, Curcumin, **8**, or **10** alone and in combination with Olaparib were profiled in wild-type zebrafish (*Danio rerio*) embryos, a model employed to assess toxicity.^48^ This was performed for both single-agent conditions, and the synergistic pairs were identified. Embryos were exposed to concentration gradients of compounds in the absence or presence of Olaparib (10 mg/L) at 1 h post fertilization (hpf). The survival and hatching rates were recorded at 24, 48, 72, and 96 hpf. Low toxicity for all three compounds was observed, with half maximal lethal concentration (LC_50_) values for all compounds greater than the maximum concentration employed (LC_50_s > 100 mg/L, Table S4 in the SI). Combination treatments tested also showed low toxicity, although the highest concentration of **8** tested (100 mg/L) alongside Olaparib (10 mg/L) resulted in 75% survival at 72 hpf onward (Figure 6a). Encouragingly, neither **10** nor Curcumin impacted the hatching rate significantly compared to the untreated control; however, **8** showed almost complete inhibition of hatching at concentrations 25 mg/L or greater, indicating embryonic toxicity (Figure 6b). Interestingly, the combination of **8** and Olaparib had improved hatching rates compared to **8** as a single agent, an unexpected result considering the respective levels of DSB damage generated by these treatment conditions in our cellular studies.

**Figure 7:**
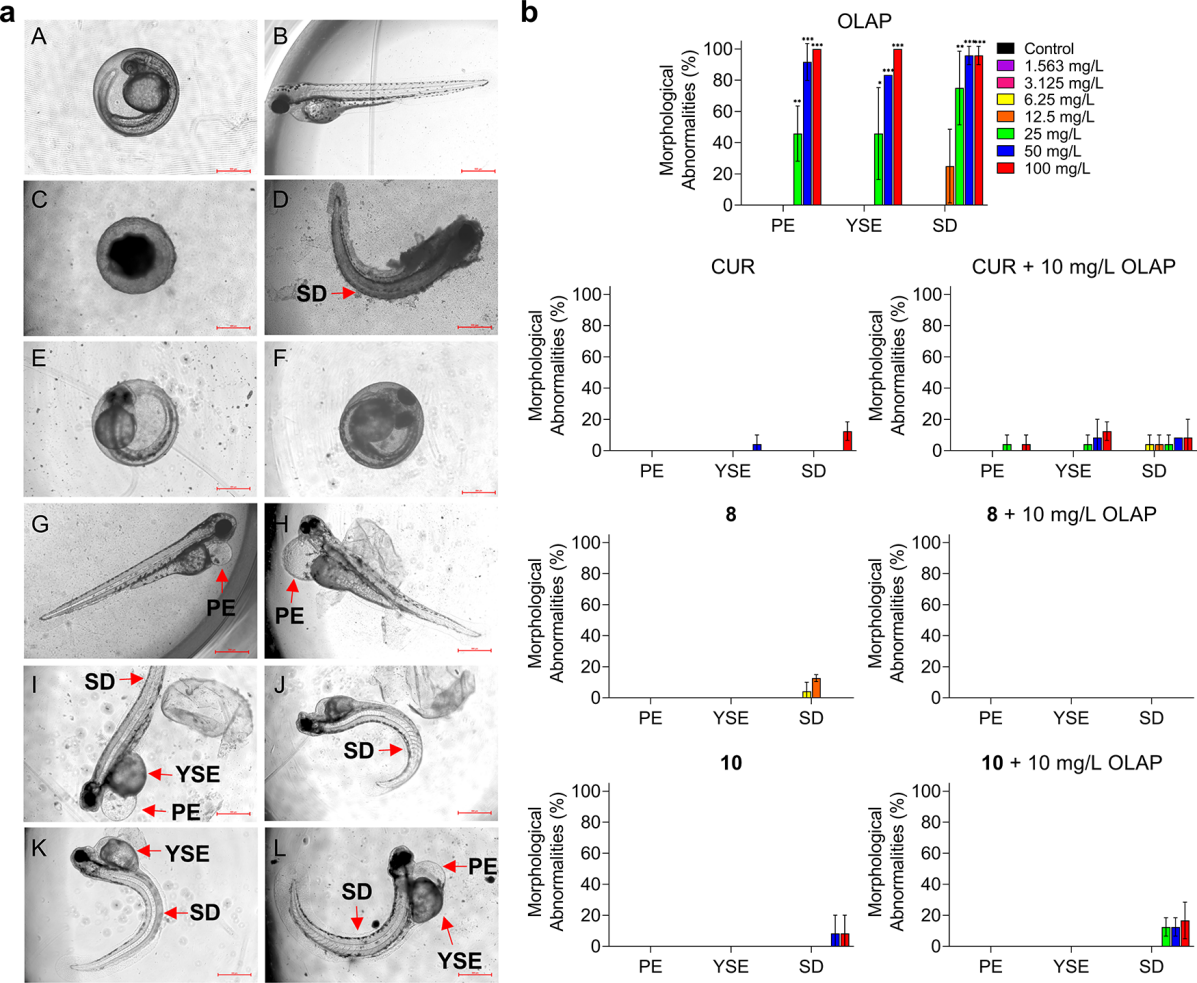
(a) Morphological assessment of zebrafish embryonic development. Normal zebrafish embryonic development at the (A) hatching stage (48 hpf) and (B) larval stage (72 hpf); (C, D) dead or coagulated embryos; (E, F) non hatching; (G–L) morphological abnormalities; precardial edema (PE), yolk sac edema (YSE), and spinal deformity (SD). Scale bars = 500 μm. (b) Morphological abnormalities of zebrafish embryos upon treatments with the stated compounds at 96 hpf. Data expressed as percentage of survival or hatching of 24 embryos from two independent experiments. **P < 0.01 and ***P < 0.001 compared to the control group by ANOVA.

The morphological changes in the development of embryos after exposure to compounds provides an indication of developmental toxicity and potential teratogenicity.^49^ Accordingly, morphological changes in the development of zebrafish embryos after exposure to Olaparib, Curcumin, **8**, or **10** were examined at 24, 48, 72, and 96 hpf (Figure 7a). These experiments show that treatment with >25 mg/L Olaparib resulted in an increase in morphological abnormalities above untreated controls in pericardial edema (PE), yolk sac edema (YSE), and spinal deformity (SD) (45.8% ± 17.7 PE; 45.8% ± 29.5 YSE; 75.0% ± 23.6 SD in 25 mg/L OLAP-treated zebrafish embryos; *P* < 0.05 for OLAP vs untreated; Figure 7b). In contrast to these results, treatment with Curcumin, **8**, or **10** did not generate significant morphological abnormalities, indicating that these compounds are not teratogenic in zebrafish at concentrations up to 100 mg/L. No substantial enhancement of morphological abnormalities was observed for Curcumin, **8**, or **10** combined with a sub-toxic concentration of Olaparib (10 mg/L), although minor increases for the Curcumin and Olaparib combination were evident (4.2% ± 5.9 PE; 12.5% ± 5.9 YSE; 8.3% ± 11.8 SD in 100 mg/L CUR + OLAP-treated zebrafish embryos; *P* > 0.05 for treated vs untreated; Figure 7b). As Curcumin and **10** show the lowest acute and developmental toxicity in the zebrafish embryo model, including in combination with Olaparib, we conclude that these two molecules are the strongest candidates for future advancement.

## Discussion and Conclusions

Examining cytotoxic potency as single agents toward TNBC cells, RPC mono-intercalators **3**, **4**, and **7** displayed comparable—or greater—activity to cisplatin. By comparing IC_50_ concentrations of the bisbipyridine complexes **1**, **2**, and **5** toward MDA-MB-231 cells, it is apparent that the selection of dmdppz as an intercalating ligand promotes greater cytotoxicity over either PIP or *p*-HPIP. Previous work has shown PIP complexes to be more cytotoxic than their dppz analogues;^39^ therefore, the order of cytotoxicity of intercalating ligands of dmdppz > *p*-HPIP > PIP > dppz can be derived. In a similar manner, by comparing IC_50_ concentrations within the [Ru(N^N)_2_(dmdppz)]^2+^ sub-series, the order of cytotoxicity for each N^N ancillary ligand can be seen to be 4,4′dmb > 5,5′dmb ∼ bpy (5,5′dmb = 5,5′-dimethyl-2,2′bipyridine, 4,4′dmb = 4,4′-dimethyl-2,2′bipyridine). While cellular internalization was not examined in the present work, both of these findings would be in agreement with improved cellular uptake facilitated by increased ligand hydrophobicity, an established concept that has been reported in numerous other studies.^28,30,39,50−52^ Along with the results of cell-free DNA binding studies, these findings also illustrate that methylated polypyridyl ligands can be employed to increase both DNA binding affinity and cytotoxicity of RPCs. It is also interesting to note that the most cytotoxic among these molecules—**3**, **4**, and **7**—showed an additive relationship with Olaparib rather than synergistic. This result likely indicates that these RPCs do not induce sufficient SSB damage or replication stress for successful PARPi combination, despite possessing reasonable anti-proliferative activities and high cell-free DNA binding affinities. This can be contrasted to our previous results for Ru-PIP and [Ru(PIP)_2_(dmb)]^2+^, each of which contains a Ru(II) center coordinated to multiple PIP or dppz ligands, where replication inhibition and subsequent Olaparib synergy were demonstrated in both cases.^31,32^ In turn, this would imply that RPCs coordinated to multiple intercalating groups are required for replication fork stalling and subsequent PARPi synergy by this mechanism of action. However, full structure–activity relationship (SAR) studies would be required to expand upon these observations.

While the distinct molecular geometries and reactivities of transition metal complexes have been highlighted as an opportunity for chemical diversity in drug discovery,^53^ they are rarely employed within chemical screening approaches. However, the potential of this approach has been demonstrated by Cohen et al., who utilized a library of 71 metallofragments as 3D scaffolds for fragment-based drug discovery.^54^ Results compared favorably to hits achieved by organic molecules, allowing the authors to conclude that metallofragments are compatible with current fragment-based drug discovery screening techniques. A meta-analysis by Frei et al. demonstrated the potential for metallocompounds as antibiotics, showing a significantly higher hit-rate (9.9%) when compared to the purely organic molecules (0.87%) in the community for the open antimicrobial drug discovery database.^55^ Although the “micro-library” employed in the present study is comparatively small, all the molecules selected for testing were required to possess high DNA binding affinities and/or previously determined DNA-damage-based cellular mechanisms of action. Combined with the mixed organic/RPC library composition, these selection criteria were chosen to increase the chances of identifying new PARPi synergistic combinations and to test the hypothesis that inclusion of metallocompounds would aid successful hit generation. It is therefore highly encouraging that our mixed organic/RPC “micro library” (containing a 53% RPC composition) successfully identified two metallocompounds along with Curcumin as hits in our synergy assay screen (a 67% RPC composition). In terms of *in vitro* Olaparib synergy, it is notable that the two metallocompounds out-performed established DNA-damaging chemotherapeutics such as cisplatin and gemcitabine and also the ATR inhibitor Ceralasertib; all of which have been examined in combination with Olaparib in clinical trials (refs (9, 11) and clinical trial identifier NCT02576444). Considering that there is evidence that Curcumin is a pan-assay interference (PAIN) compound,^56^ the two metallocompounds arguably represent the strongest hits from our proof-of-concept library, thereby providing clear justification for our mixed organic/metallocompound library design, where the discovery of new synergistic PARPi combinations was aided by the inclusion of metallocompounds within a phenotype screen.

Cellular studies have shown that the DNA-damaging agents cisplatin^57^ and gemcitabine^58^ exhibit synergy with Olaparib in cancer cell lines independent of their BRCA status. In these cases, the single-strand breaks generated by either molecule are then unrepaired due to PARP inhibition, resulting in the accumulation of DSBs and cell death by apoptosis. A similar mechanism of synergy is likely for **8** and **10**: our mechanistic studies indicate that they induce DNA damage, most notably activation of the ATR pathway in response to SSB damage or replication stress, and DSB DNA damage is then increased substantially by the addition of Olaparib, triggering elevated apoptosis. Previous studies have shown that **8** and **10** bind DNA by non-covalent interactions^41^ and cellular uptake studies have revealed an intracellular concentration three-fold greater than the external concentration.^59^ In the current study, this would correspond to intracellular concentrations in the low micromolar range achieving Olaparib synergy. When this is combined with the relatively high DNA binding affinities of the molecules (*K*_b_ = 3.3 × 10^6^ and 4.4 × 10^5^ M^–1^ for **8** and **10** respectively, Table S2) and the observed DNA damage response activation to each molecule, this strongly implies that DNA is a cellular target for each molecule and that DNA binding is responsible for resultant PARPi synergy. However, we cannot discount the possibility that the observed synergy of **8** and **10** with Olaparib is obtained by multiple mechanisms of action and/or the observed DNA damage response activation is indirect, for example, via ROS generation. Investigation into additional targets of **8** and **10** along with detailed uptake/localization studies would prove illuminating to explore the precise molecular mechanism of synergy in more detail.

It is also worth noting that **8** is also phototoxic when exposed to high light doses, generating intracellular singlet oxygen and cell-free DNA cleavage.^59^ Therefore, Olaparib synergy may be yet further enhanced by light and photoinduced DNA damage by **8**. Considering that RPCs have made substantially progress as photosensitizers for PDT,^60^ with one such agent undergoing clinical trials for bladder cancer,^61^ employing phototoxic RPCs that generate DNA damage via single oxygen or ROS generation along with DDR inhibitors could be a lucrative area of exploration. Considering that the majority of DNA damage from the ionizing radiation employed in radiotherapy are SSB breaks^62^ and PARP inhibitors function as excellent radiosensitizers due to the role of PARP enzymes in repairing SSB damage,^63^ it would be useful to similarly explore PDT photosensitizers alongside PARPi. This has the advantage that light can be employed to control the precise form of DNA damage generated and means that the dose of light required for effective phototoxicity could theoretically be reduced, thereby facilitating use of weaker light sources that have greater penetrative depth in tissue. Other studies examining RPCs as photosensitizers for PDT show that RPCs completely penetrate a HeLa cervical cancer spheroid model with a diameter of 800 μM as shown by a strong luminescence signal observed upon treatment.^64^ The fact that our identified synergistic drug combinations are effective in breast cancer spheroids, including aggressive TNBC, may be attributed to the cell penetration ability of RPCs **8** or **10**, suggesting the need for future evaluations on drug penetration of these RPCs into spheroid models.

In conclusion, a drug synergy screen identified two ruthenium(II)-rhenium(I) polypyridyl metallomacrocycles as synergistic combinations with the PARP inhibitor Olaparib in BRCA-proficient breast cancer cells, including cells with Olaparib resistance. Mechanistic studies indicated that the synergy is due to DNA damage enhancement, while the verification of action in spheroid models, low cytotoxicity toward non-malignant cells, and low zebrafish embryo toxicity make one of these candidates particularly encouraging for further development as a cancer-specific treatment in combination with Olaparib. Overall, this work supports the concept that the PARP inhibitor combination therapy represents a promising approach for cancer treatment, including toward aggressive strains such as TNBC.

## Experimental Section

### Chemistry

#### General Chemical Methods

Olaparib and Ceralasertib were purchased from MedChemExpress, Berzosertib was purchased from Abcam and Tamoxifen citrate was purchased from Tocris. All other chemicals were purchased from SigmaAldrich and ThermoFisher Scientific. All commercial reagents were used without purification unless otherwise specified. ^1^H and ^13^C NMR spectra were obtained using a Bruker Advance III 500 MHz Nuclear Magnetic Resonance Spectrometer. HRMS (high-resolution mass spectroscopy) samples were analyzed at the EPSRC UK National Mass Spectrometry Facility at Swansea University using a ThermoScientific LTQ Orbitrap XL 1 Mass Spectrometer. Fourier transform infrared spectra were run on a Perkin Elmer FT-IR Spectrometer Spectrum TWO. Elemental analysis was performed by the Elemental Analysis Service at London Metropolitan University. Analytical HPLC was carried out on the Agilent system equipped with a Waters XBridge C18 (130 Å, 3.5 μm, 4.6 × 150 mm) analytical column. Water with 0.1% trifluoroacetic acid (TFA) and acetonitrile with 0.1% TFA were used as eluents. The flow rate was 1 mL/min, and 50% A:50% B was used for 15 min. The purity of the final compounds was ≥95% as determined by HPLC or elemental microanalysis.

#### [Ru(N^N)_2_(PIP)]^2+^ Compounds

[Ru(bpy)_2_(PIP)]^2+^ (**1**), [Ru(bpy)_2_(H-PIP)]^2+^ (**2**), [Ru(phen)_2_(PIP)]^2+^ (**3**), and [Ru(phen)_2_(H-PIP)]^2+^ (**4**) (bpy = 2,2′bipyridine, phen = 1,10 phenanthroline, PIP = 2-(phenyl)-imidazo[4,5-*f*][1,10]phenanthroline, *p*-HPIP = 2-(4-hydroxyphenyl)imidazo[4,5-*f*][1,10]phenanthroline) were prepared by an adaptation of the previously reported synthetic pathway^34^ from Ru(N^N)_2_Cl_2_·2H_2_O (where N^N = bpy or phen).^65^**1**: Mass (Yield): 0.09 g (71.0%). ^1^H NMR (CD_3_CN), δ ppm: 9.08 (d, 2H, *J* = 8.0 Hz), 8.86 (d, 4H, *J* = 8.1 Hz), 8.51 (d, 2H, *J* = 8.0 Hz), 8.47 (d, 2H, *J* = 8.0 Hz), 8.27 (d, 2H, *J* = 8.0 Hz), 8.07 (t, 4H, *J* = 8.0 Hz), 7.83 (d, 2H, J = 6.0 Hz), 7.76 (t, 2H, *J* = 8.0 Hz), 7.62 (t, 2H, *J* = 6.9 Hz), 7.57 (m, 1H), 7.43 (t, 4H, *J* = 8.0 Hz). Elemental analysis (as PF_6_ salt): Calcd: C, 46.86; H, 2.82; N, 11.21; Found: C, 47.08; H, 2.87; N, 11.63. ESI-MS, *m*/*z* (%): 709.3 [M^+^], 354.6 [M^2+^].

**2**: Mass (Yield): 0.09 g (69.0%). ^1^H NMR (CD_3_CN), δ ppm: 9.06 (d, 2H, *J* = 8.0 Hz), 8.85 (d, 2H, *J* = 8.1 Hz), 8.51 (d, 2H, *J* = 8.0 Hz), 8.47 (d, 2H, *J* = 8.0 Hz), 8.12 (d, 2H, *J* = 9.2 Hz), 8.06 (t, 4H, *J* = 8.0 Hz), 7.82 (d, 2H, *J* = 5.7 Hz), 7.74 (t, 2H, *J* = 6.9 Hz), 7.42 (t, 4H, *J* = 6.9 Hz), 7.03 (d, 2H, *J* = 8.0 Hz), 5.00 (s, 1H). Elemental analysis (as PF_6_ salt): Calcd: C, 46.12; H, 2.78; N, 11.03; Found: C, 46.24; H, 2.79; N, 11.27. ESI-MS, *m*/*z* (%): 726.1 [M^+^], 363.1 [M^2+^].

**3**: Mass (Yield): 0.10 g (69.2%). ^1^H NMR (CD_3_CN), δ ppm: 9.03 (d, 4H, *J* = 8.0 Hz), 8.94 (d, 2H, *J* = 8.0 Hz), 8.57 (d, 4H, *J* = 8.0 Hz), 8.48 (d, 2H, *J* = 8.0 Hz), 8.27 (d, 2H, *J* = 6.9 Hz), 8.23 (s, 2H), 8.13 (d, 2H, *J* = 6.9 Hz), 7.99 (d, 2H, *J* = 6.9 Hz), 7.93 (d, 4H, *J* = 6.9 Hz), 7.60 (m, 1H). Elemental analysis (as PF_6_ salt): Calcd: C, 49.29; H, 2.69; N, 10.69; Found: C, 49.27; H, 2.51; N, 10.64. ESI-MS, *m*/*z* (%): 757.3 [M^+^], 379.3 [M^2+^].

**4**: Mass (Yield): 0.11 g (70.1%). ^1^H NMR (CD_3_CN), δ ppm: 9.05 (d, 4H, *J* = 6.9 Hz), 8.86 (d, 2H, *J* = 6.9 Hz), 8.52 (d, 4H, *J* = 8.0 Hz), 8.47 (d, 2H, *J* = 8.0 Hz), 8.07 (d, 4H, *J* = 8.0 Hz), 7.83 (d, 2H, *J* = 5.7 Hz), 7.57 (s, 2H), 7.43 (t, 4H, *J* = 6.9 Hz), 7.04 (d, 2H, *J* = 8.0 Hz), 5.00 (s, 1H). Elemental analysis (as PF_6_ salt): Calcd: C, 48.55; H, 2.65; N, 10.53; Found: C, 48.32; H, 2.79; N, 10.53. ESI-MS, *m*/*z* (%): 774.1 [M^+^], 387.0 [M^2+^].

#### [Ru(N^N)_2_(dmdppz)]^2+^ Compounds

[Ru(bpy)_2_(dmdppz)]^2+^ (**5**), [Ru(5,5′dmb)_2_(dmdppz)]^2+^ (**6**), and [Ru(4,4′dmb)_2_(dmdppz)]^2+^ (**7**) (dmdppz = 10,12-dimethyl-dipyrido[3,2-*a*:2′,3′-*c*]phenazine, 5,5′dmb = 5,5′-dimethyl-2,2′bipyridine, 4,4′dmb = 4,4′-dimethyl-2,2′bipyridine) were prepared by an adaptation of a previously reported synthetic pathway.^38^ This involved preparation of the [Ru(N^N)_2_(dpq)]^2+^ (dpq = 1,10-phenanthroline-5,6-dione) intermediate complex from the Ru(N^N)_2_Cl_2_·2H_2_O starting material followed by condensation with 1,2-diamino-3,5-dimethylbenzene.

Step 1: Formation of the [Ru(N^N)_2_(dpq)]^2+^ intermediate complex: The starting material Ru(N^N)_2_Cl_2_·2H_2_O (N^N = bpy, 5,5′dmb or 4,4′dmb, prepared as in ref (65)) and dpq (prepared as in ref (66)) were added to 1:1 ethanol:water. The mixture was refluxed under nitrogen gas for 3 h before being allowed to cool to room temperature (RT). 1 mL of a saturated aqueous solution of ammonium hexafluorophosphate was added, and the brown precipitate formed was collected via filtration and washed with DI water followed by ether before drying.

[Ru(bpy)_2_(dpq)]^2+^: Mass (Yield): 0.561 g (78%). ^1^H NMR (500 MHz, C_3_D_6_O), δ, ppm: 8.85 (d, *J* = 8.0 Hz, 4H), 8.65 (dd, *J* = 7.9, 1.1 Hz, 2H), 8.38 (d, *J* = 5.6 Hz, 2H), 8.25 (t, *J* = 7.9 Hz, 4H), 8.12 (m, 4H), 7.83 (dd, *J* = 7.9, 5.6 Hz, 2H), 7.60 (m, 4H). FTIR: 556, 831, 1426, 1444, 1448, and 1699 cm^–1^.

[Ru(5,5′dmb)_2_(dpq)]^2+^: Mass (Yield): 1.13 g (93%). ^1^H NMR (500 MHz, C_3_D_6_O), δ, ppm: 8.65 (d, *J* = 8.4 Hz, 4H), 8.62 (m, 2H), 8.34 (dd, *J* = 5.6, 1.2 Hz, 2H), 8.03 (d, *J* = 8.3 Hz, 4H), 7.89 (d, *J* = 35.2 Hz, 4H), 7.80 (m, 2H), 2.22 (s, 6H), 2.17 (s, 6H). FTIR: 556, 835, 1428, 1477, and 1699 cm^–1^.

[Ru(4,4′dmb)_2_(dpq)]^2+^: Mass (Yield): 0.708 g (89%). ^1^H NMR (500 MHz, C_3_D_6_O), δ, ppm: 8.69 (s, 4H), 8.60 (d, *J* = 6.9 Hz, 2H), 8.34 (m, 2H), 7.94 (d, *J* = 5.8 Hz, 2H), 7.86 (d, *J* = 5.8 Hz, 2H), 7.79 (m, 2H), 7.42 (d, *J* = 5.9 Hz, 2H), 7.38 (d, *J* = 5.4 Hz, 2H), 2.58 (s, 6H), 2.56 (s, 6H). FTIR: 557, 826, 1427, and 1704 cm^–1^.

Step 2: 1,2-Diamino-3,5-dimethylbenzene was added to [Ru(N^N)_2_(dpq)](PF_6_)_2_ in hot, anhydrous methanol in a 4.5:1 molar ratio. The mixture was refluxed for 6 h under nitrogen and allowed to cool to RT. A saturated aqueous solution of ammonium hexafluorophosphate was added, and the mixture was cooled on ice. The bright orange precipitate was collected by filtration and recrystallized from acetonitrile and ether. The crystals formed were collected by filtration, washed with ether, and dried.

**5**: Mass (Yield): 0.164 g (70%). ^1^H NMR (500 MHz, C_3_D_6_O), δ, ppm: 9.82 (s, 1H), 9.75 (d, *J* = 7.1 Hz, 1H), 8.88 (d, *J* = 9.5 Hz, 4H), 8.54 (dd, *J* = 7.7, 2.7 Hz, 2H), 8.29 (s, 2H), 8.15 (m, 9H), 7.90 (s, 1H), 7.67 (s, 2H), 7.43 (d, *J* = 1.4 Hz, 2H), 3.03 (s, 3H), 2.72 (s, 3H). ^13^C NMR (126 MHz, C_3_D_6_O), δ, ppm: 157.5, 157.3, 153.7, 153.5, 152.3, 150.6, 143.7, 143.3, 138.1, 137.6, 134.6, 133.6, 127.9, 127.8, 127.6, 125.9, 124.5, 21.33, 16.30. HRMS for RuC_40_H_30_N_8_P_2_F_12_: [M – 2PF_6_]^2+^ at 362.0814 and [M – PF_6_]^+^ at 869.1272. FTIR: 557, 836, and 1447 cm^–1^. Elemental analysis for [**5**](Cl)_2_·6H_2_O, C4_0_H_42_N_8_RuCl_2_O_6_: Calc’d: C, 53.2; H, 4.7; N, 12.4. Found: C, 52.5; H, 5.2; N, 11.0.

**6**: Mass (Yield): 0.233 g (87%). ^1^H NMR (500 MHz, C_3_D_6_O), δ, ppm: 9.78 (d, *J* = 7.2 Hz, 1H), 9.70 (m, 1H), 8.69 (d, *J* = 8.4 Hz, 2H), 8.65 (d, *J* = 8.3 Hz, 2H), 8.60 (t, *J* = 6.4 Hz, 2H), 8.49 (dd, *J* = 6.0, 2.7 Hz, 2H), 8.07 (d, *J* = 6.8 Hz, 3H), 7.97 (s, 1H), 7.87 (s, 2H), 7.23 (s, 2H), 7.18 (s, 2H), 3.03 (s, 3H), 2.71 (s, 3H), 2.27 (s, 6H), 2.02 (s, 6H). ^13^C NMR (126 MHz, C_3_D_6_O), δ, ppm: 155.3, 154.8, 153.8, 153.5, 152.0, 151.7, 138.7, 138.6, 138.3, 134.5, 133.3, 127.4, 125.7, 123.4, 123.3, 122.9, 21.34, 17.6, 17.5, 17.4, 16.30. HRMS for RuC_44_H_38_N_8_Cl_2_: [M – 2Cl]^2+^ at 390.1123. FTIR: 557, 837, and 1477 cm^–1^. Elemental analysis for [**6**](Cl)_2_·6H_2_O, C_44_H_50_Cl_2_N_8_O_6_Ru: Calc’d: C, 55.1; H, 5.3; N, 11.7. Found: C, 53.0; H, 5.4; N, 11.1.

**7**: Mass (Yield): 0.544 g (81%). ^1^H NMR (500 MHz, C_3_D_6_O), δ, ppm: 9.76 (d, *J* = 7.6 Hz, 1H), 9.69 (d, *J* = 8.1 Hz, 1H), 8.74 (s, 2H), 8.70 (s, 2H), 8.51 (m, 2H), 8.08 (d, *J* = 6.2 Hz, 2H), 8.05 (m, 2H), 7.98 (dd, *J* = 5.7, 1.8 Hz, 2H), 7.88 (d, *J* = 2.8 Hz, 2H), 7.48 (d, *J* = 5.6 Hz, 2H), 7.23 (d, *J* = 5.9 Hz, 2H), 3.02 (s, 3H), 2.71 (s, 3H), 2.62 (s, 6H), 2.51 (s, 6H). ^13^C NMR (126 MHz, C_3_D_6_O), δ, ppm: 156.9, 154.6, 154.5, 151.4, 151.1, 150.4, 150.3, 137.1, 134.8, 133.2, 128.6, 128.4, 127.4, 125.8, 125.11, 125.03, 21.32, 20.3, 20.2, 16.3. HRMS for RuC_44_H_38_N_8_Cl_2_: [M – 2Cl]^2+^ at 390.1130. FTIR: 556, 840, and 1414 cm^–1^. Elemental analysis for [**7**](PF_6_)_2_, C_44_H_38_N_8_RuP_2_F_12_: Calc’d: C, 49.4; H, 3.6; N, 10.4. Found: C, 47.9; H, 3.6; N, 10.1.

#### Ru Macrocycles

Ru(*bpy*)Re (**8**), Ru(phen)Re (**9**), and Ru(dppz)Re (**10**) were synthesized and characterized as reported previously.^36,37^

#### DNA Binding Studies

Luminescence titrations were carried out with the addition of an aqueous solution of concentrated calf thymus DNA in aqueous Tris buffer (25 mM NaCl, 5 mM Tris, pH = 7) to 3 μM **5**, **6**, and **7**. After each addition of DNA, the solution was mixed by a pipette and allowed to equilibrate for 2 min. The spectra were recorded on a Perkin Elmer Fluorescence Spectrometer LS 55. At least 20 data points before the emission intensity reached a maximum were obtained. The luminescence emission spectra were obtained using an excitation wavelength of 450 nm, and the emission intensities were measured from 500 to 800 nm. The AUC (area under the curve) for each spectrum were used to generate Scatchard plots and fit to the McGhee von Hippel binding model,^67^ in which neither the site size nor binding constant was defined, to determine *K*_b_ and *n*. The binding constant of **3** with calf-thymus DNA was determined from UV–Vis titrations, as described by Liu et al.^34^

### Biology

#### Reagents

Antibodies for p-Chk1 (Ser345), p-ATR (Thr1989), total ATR, p-ATM (Ser 1981), total ATM, p-histone H2AX (Ser139), β-actin, and HRP-linked secondary antibody were purchased from Cell Signaling Technology (CST). Antibodies for PARP1, total Chk1, and Alexa-Fluor 488 conjugated secondary antibodies were purchased from Abcam. Antibodies for PARG and P-gp were purchased from Santa Cruz Biotechnology and Elabscience, respectively. All ruthenium(II) compounds were converted to their chloride salts by anion metathesis. Stock solutions of all compounds—except cisplatin—were prepared at 100 mM in 100% dimethyl sulfoxide (DMSO) and further diluted using Dulbecco’s modified Eagle’s medium (DMEM). The final DMSO concentration employed in the cell studies was 0.1%. Stock solutions of cisplatin (2 mM) were prepared in phosphate buffered saline (PBS).

#### Cell Culture

MCF7 and MDA-MB-231 breast cancer cell lines were cultured in DMEM supplemented with 10% fetal bovine serum (FBS) and 1% penicillin/streptomycin antibiotic. The MCF10A normal breast cell line was cultured in DMEM supplemented with 5% horse serum, 0.5 μg/mL hydrocortisone, 20 ng/mL recombinant human EGF (hEGF), 10 μg/mL insulin, and 1% penicillin/streptomycin antibiotic. Cells were maintained at 37 °C under a humified atmosphere containing 5% CO_2_ and routinely subcultured with trypsin.

#### Generation of Olaparib-Resistant MDA-MB-231 Cells

The Olaparib-resistant MDA-MB-231 cell line (MDA-MB-231R) was developed from the parental MDA-MB-231 cell line by long-term drug exposure (∼8 months, 10–100 μM Olaparib) in a similar manner described by Kim et al.^45^ MDA-MB-231 cells were seeded at a density of 1.5 × 10^5^ cells in a 25 cm^2^ flask and allowed to adhere for 24 h. Cells were treated with a starting Olaparib concentration of 10 μM, and treatments were refreshed every 3 days. After each week, the confluency was assessed: if confluency is <50%, the treatment was stopped; if confluency is at 50–70%, the treatment was maintained; and if confluency is over 70%, a portion of the cells was frozen. When the cells adapted to a new drug concentration, cells were reseeded at a density of 1.5 × 10^5^ cells in a new 25 cm^2^ flask and allowed to adhere before the next exposure to the increased concentration of Olaparib. This procedure was repeated to achieve a final concentration of Olaparib of 100 μM. Following this, MDA-MB-231R cells were maintained in a low concentration of Olaparib (10 μM) to preserve resistance. The MTT assay and clonogenic survival assay were conducted to monitor the sensitivity of resistant cells. Prior to any downstream studies, MDA-MB-231R cells were grown without Olaparib treatments for a single passage.

#### MTT Assay

Cells were seeded at 5 × 10^3^ cells/well for MCF7 cells and 1 × 10^4^ cells/well for MDA-MB-231 and MCF10A cells, respectively, in 96-well plates, allowed to adhere for 24 h, and treated as described in the main text. Following treatment, solutions were removed, thiazolyl blue tetrazolium bromide (MTT, 0.5 mg/mL) reagent was added to the cells, and plates were incubated for 4 h. The reduced purple formazan crystals were solubilized with 100 μL of DMSO, and the absorbance at 570 nm (620 nm as reference wavelength) was measured using a microplate reader. The average in percent reduction of cell viability was expressed relative to untreated control cells. The combinatorial effect was evaluated by the generation of the compound dose–response curve and a shift of the IC_50_ value in the presence or absence of Olaparib (graphed and calculated using GraphPad Prism software).

#### Drug Interaction Analysis

Dose–effect curves for single agents and their combinations were generated from the MTT assay data, and the combination index (CI) values were calculated using CalcuSyn and CompuSyn software (Biosoft, Cambridge, UK) as established by Chou and Talalay.^43^ CI < 0.9 indicates synergism, CI = 0.9–1.0 indicates additive, and CI > 1 indicates antagonism. GraphPad Prism Software was used to generate a 3-color scale based on CI values obtained, where synergism is represented by green, additive by yellow, and antagonism by red. The colors of each CI value were interpolated in between these constraints accordingly.

#### Clonogenic Survival Assay

Cells were seeded at 1 × 10^3^ cells/well in 6-well plates, allowed to adhere for 24 h, and treated as described in the main text. After treatment, solutions were removed, and cells were cultured in compound-free medium for 7–10 days to allow colony formation. Cells were then washed (1× PBS, twice), fixed (ice-cold 100% methanol, 15–20 min, 4 °C), and stained (0.5% crystal violet solution, 20 min). The staining solution was washed with water, and images were photographed with a digital camera. Individual colonies were counted using ImageJ software, and the survival fraction was determined (normalized to controls).

#### Cell Cycle Analysis

Cells were seeded at 3 × 10^5^ cells/well in 6-well plates, allowed to adhere for 24 h, and treated as described in the main text. Following treatment, cells were trypsinized and washed with 1× PBS twice. This was followed by fixation in ice-cold 70% ethanol for at least overnight at 4 °C. After fixation, fixed cells were centrifuged (1000 rpm, 5 min), and the resulting cell pellets were washed with 1× PBS twice. Samples were resuspended in 500 μL of 1× PBS, treated with RNase A solution (5 μL, 10 mg/mL, 15 min), and stained with propidium iodide (PI) (2 μL, 5 mg/mL, in the dark). Thereafter, samples were acquired and analyzed with a NovoCyte flow cytometer (Agilent Technologies) and NovoExpress software. For each sample, a minimum of 10,000 cells were counted.

#### Apoptosis Annexin V-FITC/PI Assay

Cells were seeded at 3 × 10^5^ cells/well in 6-well plates, allowed to adhere for 24 h, and treated as described in the main text. After treatment, cells were trypsinized and washed with 1× PBS twice. This was followed by the addition of 500 μL of 1× binding buffer and 5 μL of Annexin V-FITC (Invitrogen). The cell-containing mixture was incubated for 20 min at RT. A total of 5 μL of PI (20 μg/mL) was added prior to flow cytometric analysis using a flow cytometer, and results were analyzed using NovoExpress software. For each sample, a minimum of 10,000 cells were counted.

#### Determination of Reactive Oxygen Species (ROS) Levels

Cells were seeded in 6-well plate at density of 1 × 10^5^ cells/well, allowed to adhere for 24 h, and incubated with 10 μM 2′,7′-dichlorofluorescein diacetate (DCFDA) in serum-free culture media for 30 min at 37 °C in the dark. Upon completion, DCFDA solutions were removed, cells were washed with 1× PBS twice, and treated as described in the main text. Following incubation, resultant cells were harvested, washed with 1× PBS twice, and resuspended in 1× PBS. The intensity of the formed 2′7′-dichlorofluorescein (DCF) as a result of carboxy-DCFDA hydrolysis by intracellular ROS was analyzed using a flow cytometer and NovoExpress software at an excitation and emission wavelength of 488 nm and 525 nm, respectively. For each sample, a minimum of 10,000 cells were counted.

#### γH2AX Immunostaining

Cells were seeded at 3 × 10^5^ cells/well in 6-well plates, allowed to adhere for 24 h, and treated as described in the main text. Following treatment, cells were trypsinized and washed with 1× PBS twice. Cells were then fixed with 4% paraformaldehyde (PFA) for 15 min at RT. Following fixation, cells were washed with 1× PBS and resuspended in 500 μL of 1× PBS. Thereafter, cells were permeabilized by adding ice-cold 100% methanol slowly to pre-chilled cells while gently vortexing to a final concentration of 90% methanol and left on ice for 10 min. Cells were then washed in excess 1× PBS and incubated with diluted primary antibody (γH2AX) for 1 h at RT. Following incubation, cells were washed with antibody dilution buffer and incubated with diluted fluorochrome-conjugated secondary antibody (30 min, RT, in the dark). Thereafter, samples were washed with antibody dilution buffer and resuspended in 500 μL of 1× PBS. Samples were acquired and analyzed with a NovoCyte flow cytometer and NovoExpress software. For each sample, a minimum of 10,000 cells were counted.

#### Alkaline Comet Assay

Cells were seeded in 24-well plates at a density of 1 × 10^5^ cells/well, allowed to adhere for 24 h, and treated as described in the main text. Following incubation, cells were harvested and resuspended in ice-cold 1× PBS. A total of 16 μL of cell suspension was mixed with 160 μL of 1% low melting agarose (1/10 ratio; v/v). A total of 80 μL of cell suspension was immediately dropped onto the pre-coated agarose slides (1% normal melting agarose) and covered with coverslips, and slides were cooled (15 min, 4 °C, in the dark). Thereafter, coverslips were removed, and slides were immersed in pre-chilled lysis buffer (2.5 mM NaCl, 100 mM Na_2_EDTA, 100 mM Tris–HCl, and 1.6 g of NaOH; pH 10) for 2 h at 4 °C in the dark. Thereafter, slides were immersed in pre-chilled alkaline solution (1 mM Na_2_EDTA and 300 mM NaOH; pH > 13) for 1 h at 4 °C in the dark. Electrophoresis was conducted in a chamber filled with pre-chilled alkaline electrophoresis solution (300 mM NaOH and 1.0 mM EDTA; pH > 13) under standard conditions (22 V; 300 mA; 1 V/cm) for 30 min in the dark. Slides were then neutralized with neutralization buffer (0.4 M Tris–HCl, pH 7.5) for 10 min, washed (ice-cold water, 2×, 10 min each), fixed (ice-cold 70% ethanol, 5 min), and air-dried for 15–30 min. Thereafter, slides were stained with 5 μg/mL Hoechst 33342 solution (30 min, RT, in the dark) and imaged using a fluorescence microscope (Zeiss Axio Vert.A1). At least 50–100 cells were analyzed per treatment. The percentage of DNA in the tail was used as a parameter of DNA damage.

#### Immunoblotting

Cells were treated as described in the main text. After treatment, cells were washed with ice-cold 1× PBS and lysed in RIPA (radioimmunoprecipitation assay) buffer containing protease inhibitors and phosphatase inhibitors. Aliquots of cell lysates (20–40 μg of total protein) were resolved by 4–20% Mini-PROTEAN TGX precast protein gels, transferred onto a nitrocellulose membrane, and probed with primary antibodies in 5% BSA (bovine serum albumin) in TBS-T (0.1% Tween 20 in 1× TBS) solutions. Reactions were visualized with a suitable horseradish peroxidase (HRP)-conjugated secondary antibody. Signal Fire ECL reagent (CST) or WesternBright ECL HRP substrate (Advansta) chemiluminescent substrates with a Syngene G:Box gel documentation system were used to visualize protein expression. ImageJ software was used for densitometry data acquisition.

#### Spheroid Growth Studies

Spheroids were grown using a liquid overlay technique. Cells were seeded in agarose-coated (50 μL, 0.6%) 96-well plates at 2000, 4000, 6000, 8000, and 10,000 cells/well and incubated for 15 days at 37 °C such that each well contained a single spheroid. Formation of spheroids was observed every three days, and spheroids were imaged using a microscope attached to a digital camera at days 3, 6, 9, 12, and 15. During growth, 50% of the media was exchanged every two to three days. The volume and diameter of spheroids were determined by measuring their cross-sectional area using ImageJ software (data not shown).

#### Spheroid Growth Inhibition Studies

Spheroids were grown as described initially. The initial cell seeding density was chosen such that spheroids reached a diameter of about 400–500 μM after 3 days (4000 cells/well and 8000 cells/well for MDA-MB-231 and MCF7, respectively). Spheroids were treated with the identified combinations and single drugs alone for 72 h. Every other day thereafter, 50% of the treatment-containing medium was replaced. Spheroids were imaged every three days for a period of 12 days using a microscope attached to a digital camera. The structural integrity of spheroids following treatments was observed, and the volume was measured as initially described.

#### Spheroid Live/Dead Staining

Spheroids were grown in agarose-coated (172 μL, 0.6%) 48-well plates. Spheroids were then incubated with the identified combinations and single drugs alone for 72 h by replacing 50% of the medium with treatment-containing media. Following this, half of the culture media was replaced with staining solutions at 2× of their final concentrations for 30 min at 37 °C in the dark. The final concentrations used were 1 μM, 5 μg/mL, and 2 μg/mL for Calcein AM (Abcam), Hoechst 33342, and PI in 1× PBS, respectively. Next, spheroids were washed with 1× PBS and fixed with 4% PFA for 30 min at RT. After fixation, spheroids were washed with 1× PBS twice and the triple-stained spheroids were imaged using a fluorescence microscope (Zeiss Axio Vert.A1) to evaluate cellular viability.

#### Zebrafish (ZF) Embryo Toxicity

The zebrafish embryo toxicity study was conducted according to the guidelines for care and use of Animal Biochemistry & Biotechnology Laboratory, Faculty of Biotechnology and Biomolecular Sciences, Universiti Putra Malaysia (UPM), which has been approved by the Institutional Animal Care and Use Committee (IACUC) of UPM (UPM/IACUC/AUP No. R059/2018). Wild-type zebrafish embryos were obtained from breeding facilities at Danio Assay Laboratories Sdn.Bhd. (Universiti Putra Malaysia). Newly fertilized eggs at less than 1 hpf were collected and washed with deionized water and incubated at RT (28 ± 1 °C) in Danio-SprintM embryo media containing 0.1% DMSO. Embryos were then transferred into 96-well plates (one embryo/well) and exposed to concentration gradients of compound(s) (1.56 to 100 mg/L) alone or in combination with 10 mg/L Olaparib. Danio-SprintM embryo media were used as a control. Survival and hatching rates were observed under a microscope attached with a digital camera and imaged at 24, 48, 72, and 96 hpf. Morphological changes in the development of ZF embryos after exposure to compounds (pericardial edema, yolk sac edema, and spinal deformity) were also observed. Four lethal endpoints were evaluated including coagulated embryos, lack of somite formation, non-detachment of the tail, and lack of heartbeat. All these characteristics were recorded every 24 hpf, except the heartbeat, which is visible after 48 hpf. The LC_50_ values, known as the concentration of compound(s) that causes death of 50% of zebrafish embryo/larvae, were determined by using the GraphPad Prism software.

#### Statistical Analysis

Statistical analysis of the data was carried out using GraphPad Prism software in which the data obtained was analyzed using Student’s *t*-test or one-way analysis of variance (ANOVA). The differences between the groups were considered significant when *P* values generated were less than 0.05.

## Supporting Information Available

Details on commercially available compounds employed; DNA binding parameters of RPCs with DNA; IC_50_ values toward MDA-MB-231 and MCF7 cells, as determined by the clonogenic survival assay; LC_50_ concentrations toward zebrafish embryos; NMR, FT-IR, ESI-MS spectra, and HPLC chromatograms of novel compounds **5**, **6**, and **7**; cell viability graphs; additional Western blot data of DNA damage response activation; ROS levels of MDA-MB-231 and MCF7 cells; γH2AX levels in MCF7 cells; cell doubling time and validation of Olaparib-resistant MDA-MB-231R cells; full Western blots (PDF)
Compound structures in SMILES string notation with tabulated cell viability data (CSV)

## Abbreviations Used

4,4′dmb: 4,4′-dimethyl-2,2′bipyridine
5,5′dmb: 5,5′-dimethyl-2,2′bipyridine
5FU: fluorouracil
ATM: ataxia-telangiectasia mutated
ATR: ataxia telangiectasia and Rad3-related protein
bpy: 2,2′bipyridine
CI: combination index
CUR: Curcumin
DCFDA: 2′,7′-dichlorofluorescein diacetate
DDR: DNA damage response
dmdppz: 10,12-dimethyl-dipyrido[3,2-*a*:2′,3′-*c*]phenazine
DMSO: dimethyl sulfoxide
DNA: deoxyribonucleic acid
DMEM: Dulbecco’s modified Eagle’s medium
dppz: dipyrido[3,2-*a*:2′,3′-*c*]phenazine
DSB: double-strand break
EDTA: ethylenediaminetetraacetic acid
EGF: epidermal growth factor
FITC: fluorescein isothiocyanate
FT-IR: Fourier transform infrared spectroscopy
H-PIP: (2-(4-hydroxyphenyl)imidazo[4,5-*f*][1,10]phenanthroline)
HPLC: high-performance liquid chromatography
HRMS: high-resolution mass spectrometry
HRP: horseradish peroxidase
MTT: 3-(4,5-dimethylthiazol-2-yl)-2,5-diphenyltetrazolium bromide
OLAP: olaparib
PARG: poly(ADP-ribose) glycohydrolase
PARP: poly(ADP-ribose) polymerase
PARPi: PARP inhibitor
PBS: phosphate buffered saline
PE: precardial edema
P-gp: p-glycoprotein
phen: 1,10 phenanthroline
*p*-HPIP: 2-(4-hydroxyphenyl)imidazo[4,5-*f*][1,10]phenanthroline
PI: propidium iodide
PIP: 2-(phenyl)-imidazo[4,5-*f*][1,10]phenanthroline
qtpy: 2,2′:4,4″:4′,4″′ quaterpyridyl
RIPA: radioimmunoprecipitation assay
ROS: reactive oxygen species
RPC: ruthenium(II) polypyridyl complex
RT: room temperature
SD: spinal deformity
SI: selectivity index
SSB: single-strand break
TFA: trifluoroacetic acid
Tris: trisaminomethane
TNBC: triple-negative breast cancer
YSE: yolk sac edema
