# Supplementary material for: Discovery of Ruthenium(II) Metallocompound and Olaparib Synergy for Cancer Combination Therapy
Source: J Med Chem. 2023 May 15;66(10):6922–37. doi: 10.1021/acs.jmedchem.3c00322 (PMC10226041; doi:10.1021/acs.jmedchem.3c00322)
Supplement: Supplementary file 1 — jm3c00322_si_001.pdf [file jm3c00322_si_001.pdf]

Supporting Information for:

Discovery of ruthenium(II) metallocompound and Olaparib synergy for cancer combination therapy

Nur Aininie Yusoh,[a] Paul R. Tiley,[b] Steffan D. James,[b] Siti Norain Harun,[c] Jim A. Thomas,[d] Norazalina Saad,[a] Ling-Wei Hii,[e] Suet Lin Chia,[a,f] Martin R. Gill,*[b] and Haslina Ahmad*[a,c]

a UPM-MAKNA Cancer Research Laboratory, Institute of Bioscience, Universiti Putra Malaysia, 43400 UPM Serdang, Selangor, Malaysia

b Department of Chemistry, Faculty of Science and Engineering, Swansea University, Swansea, SA2 8PP, UK

c Department of Chemistry, Faculty of Science, Universiti Putra Malaysia, 43400 UPM Serdang, Selangor, Malaysia

d Department of Chemistry, University of Sheffield, Sheffield, S3 7HF, UK

e Center for Cancer and Stem Cell Research, Development and Innovation (IRDI), Institute for Research, International Medical University, Kuala Lumpur 57000, Malaysia

f Department of Microbiology, Faculty of Biotechnology and Biomolecular Science, Universiti Putra Malaysia, 43400 UPM Serdang, Selangor, Malaysia

email: m.r.gill@swansea.ac.uk or haslina_ahmad@upm.edu.my

Contents

Supplementary Tables… p2

Supplementary Figures… p4

References… p23

Supplementary Tables

Table S1. Details on commercially-available compounds employed. Log P = octanol/water partition coefficient.

Compound	Mol weight (g/mol)	Log P	Lipinski Y/N	FDA approved Y/N	Cellular DNA damage or target?	Cat. No/Reference
Olaparib	434.46	1.96	Y	Y	PARP inhibitor	CAS 763113-22-0
Cisplatin	301.10	-2.19	N	Y	DNA platinator	CAS 15663-27-1
Gemcitabine hydrochloride	263.20	-1.50	Y	Y	DNA replication inhibitor	CAS 122111-03-9
Fluorouracil	130.08	-0.66	Y	Y	DNA replication inhibitor	CAS 51-21-8
Tamoxifen citrate	371.51	6.35	N	Y	Ref [1]	CAS 54965-24-1
Berzosertib	463.56	3.16	Y	N	ATR inhibitor	CAS 1232416-25-9
Ceralasertib	412.51	1.91	Y	N	ATR inhibitor	CAS 1352226-88-0
NU1025	176.71	0.47	Y	N	PARP inhibitor	CAS 90417-38-2
Quercetin	302.24	2.16	Y	N	DNA intercalator (ref [2])	CAS 117-39-5
Curcumin	368.38	4.12	Y	N	Ref [3]	CAS 458-37-7

Table S2. Selected binding parameters of RPCs with DNA. K_b = DNA binding constant, n = binding site size in base pairs (bp). **3** determined by UV-Visible titration, **5**-**7** determined by luminescence titrations.

Compound	DNA K_b /M^{-1}	*n*/bp	Ref
$[Ru(bpy)_2(PIP)]^{2+}$ (**1**)	4.7×10^5	ND	[4]
$[Ru(bpy)_2(H\text{-}PIP)]^{2+}$ (**2**)	6.5 x 10^5	ND	[4]
$[Ru(phen)_2(PIP)]^{2+}$ (**3**)	6.7×10^5	ND	This work
$[Ru(phen)_2(H\text{-}PIP)]^{2+}$ (**4**)	6.9×10^4	ND	[5]
$[Ru(bpy)_2(dmdppz)]^{2+}$ (**5**)	8.7×10^6	1.6	This work
$[Ru(5,5'dmb)_2(dmdppz)]^{2+}$ (**6**)	9.2×10^6	1.4	This work
$[Ru(4,4'dmb)_2(dmdppz)]^{2+}$ (**7**)	5.7×10^6	0.7	This work
Ru(bpy)Re (**8**)	3.3×10^6	1.1	[6]
Ru(phen)Re (**9**)	8.8×10^5	4.7	[7]
Ru(dppz)Re (**10**)	4.4×10^5	1.4	[7]

Table S3. Half inhibitory IC_{50} values of Olaparib in the presence and absence of the stated single-agent (1 µM) towards MDA-MB-231 and MCF7 cells, as determined by clonogenic survival assay. 72 h treatment.

**Cell Lines**	**Compound**	**IC_{50} values of OLAP (µM)**		**Fold shift**
		**- Compound**	**+ Compound**	
MDA-MB-231	Curcumin	>10.00	0.01 ± 0.01	>1000
	**8**		0.07 ± 0.03	>142.9
	**10**		0.05 ± 0.02	>200
MCF7	Curcumin	2.72 ± 0.45	0.06 ± 0.01	45.3
	**8**		0.09 ± 0.01	30.2
	**10**		0.57 ± 0.02	4.8

Table S4. LC_{50} concentrations of Olaparib, Curcumin, **8** and **10** following 96 h treatments on zebrafish embryos.

**Compound**	**96 hpf LC_{50} (mg/L)**	**96 hpf LC_{50} (µM)**
OLAP	>100	>230.2
Curcumin	>100	>271.5
Curcumin + OLAP	>100	>271.5
**8**	>100	>45.4
**8** + OLAP	>100	>45.4
**10**	>100	>37.0
**10** + OLAP	>100	>37.0

Supplementary Figures

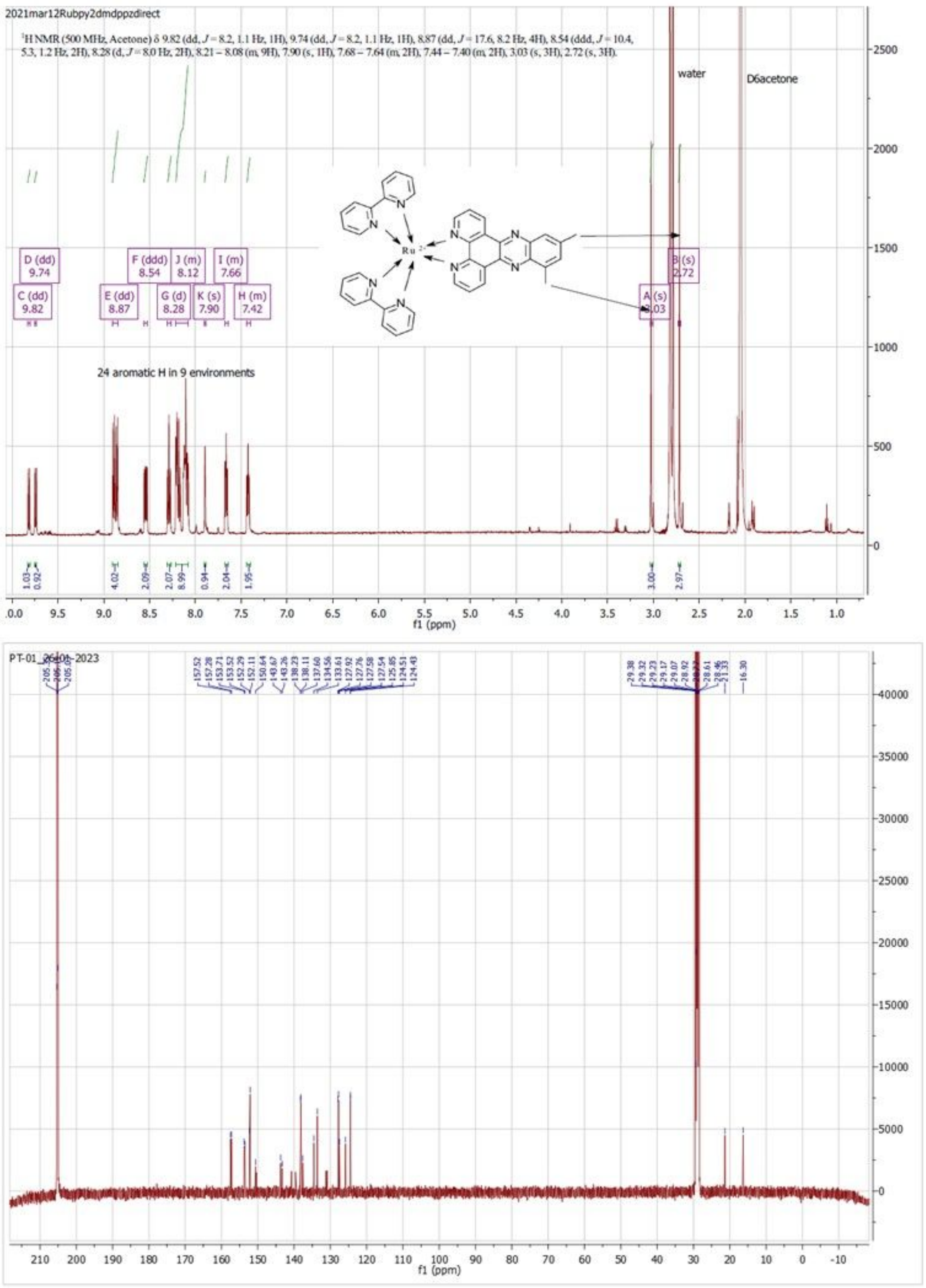

Figure S1. ^{1}H NMR (500 MHz, $(CD_3)_2CO$) (top) and ^{13}C NMR (126 MHz, $(CD_3)_2CO$) (bottom) spectra of **5**.

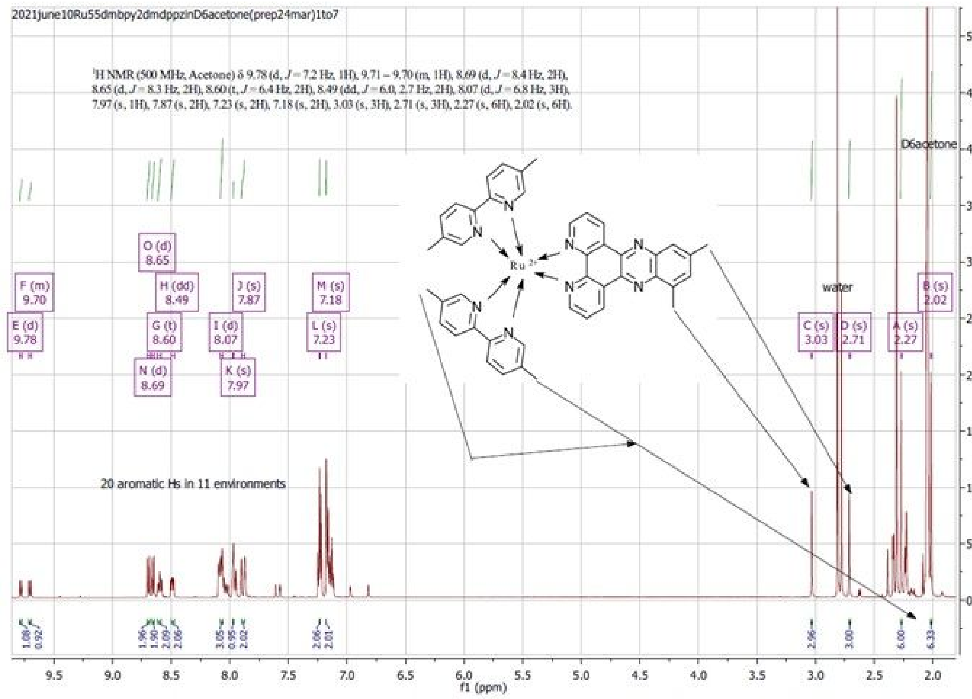

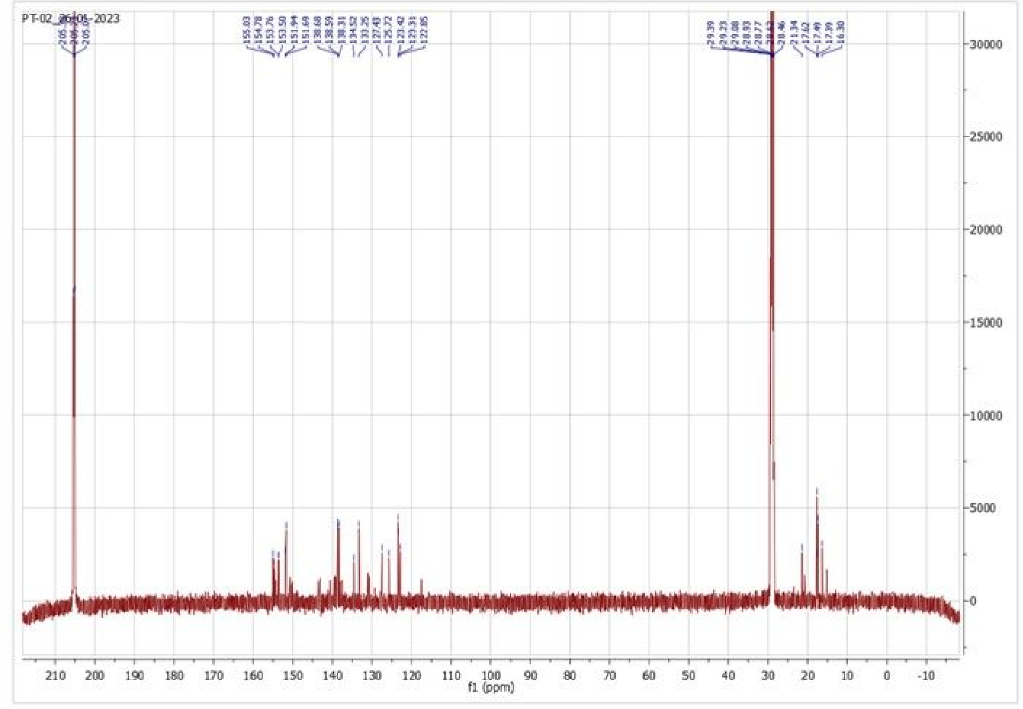

Figure S2. ^{1}H NMR (500 MHz, $(CD_3)_2CO$) (top) and ^{13}C NMR (126 MHz, $(CD_3)_2CO$) (bottom) spectra of **6**.

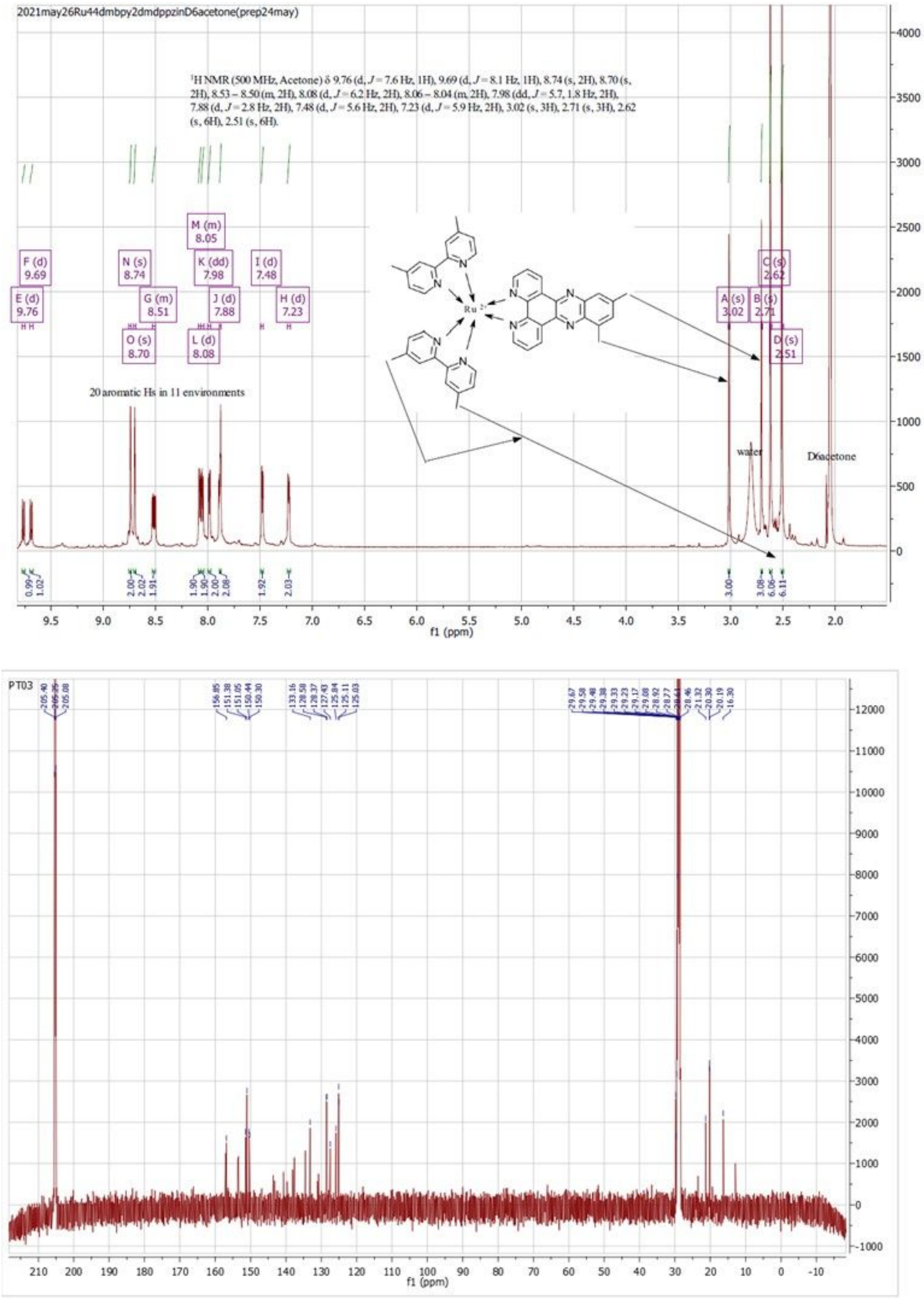

Figure S3. ^{1}H NMR (500 MHz, $(CD_3)_2CO$) (top) and ^{13}C NMR (126 MHz, $(CD_3)_2CO$) (bottom) spectra of **7**.

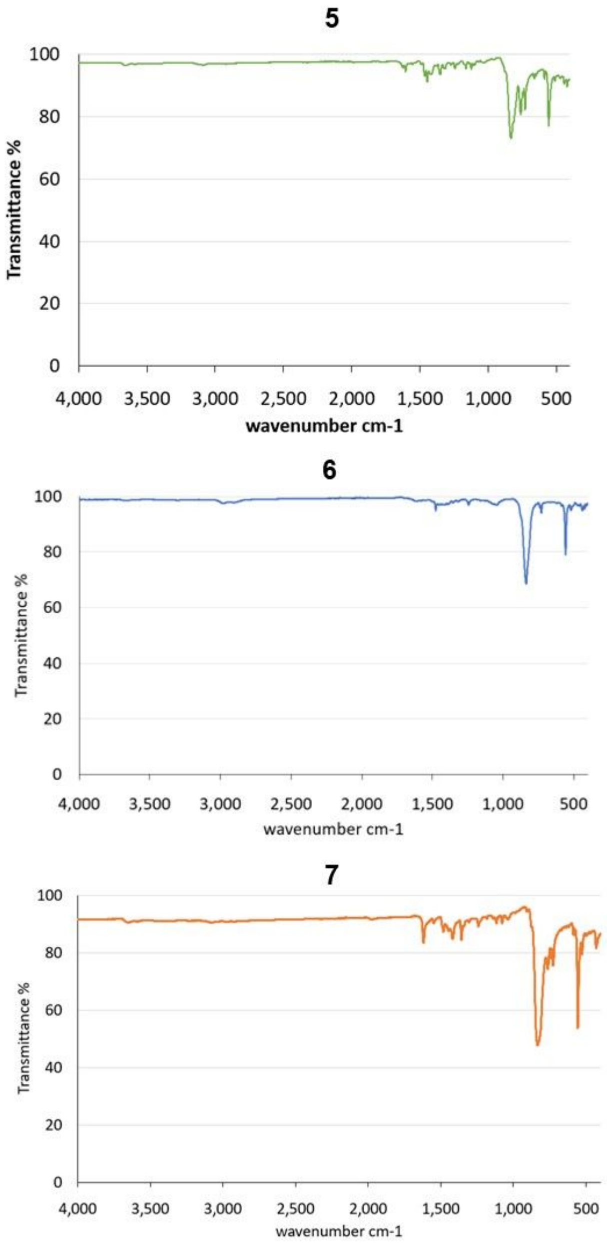

Figure S4. FT-IR spectra of **5**, **6** and **7**.

a

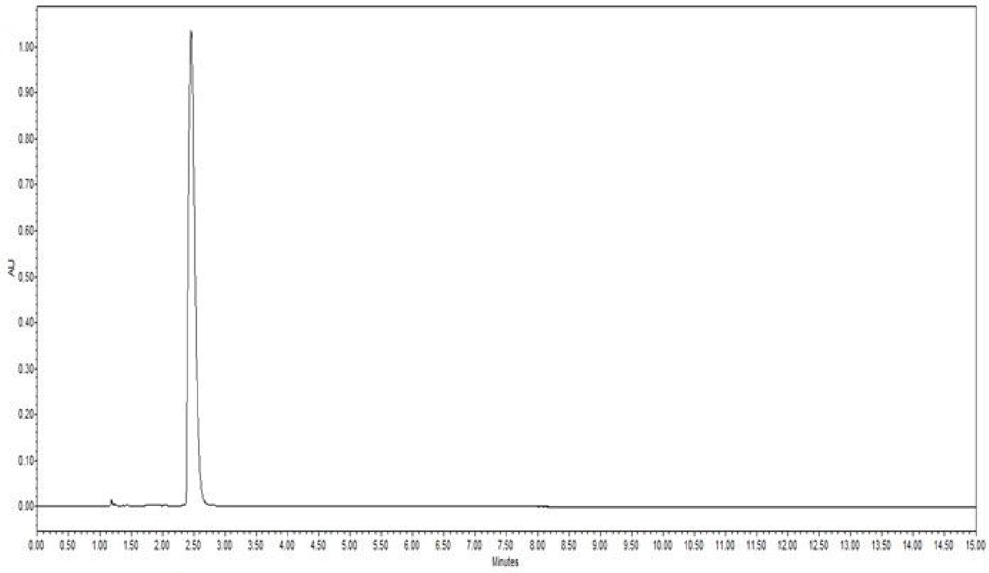

	Name	Retention Time (min)	Area (µV*sec)	% Area	Height (µV)	Int Type	Amount	Units	Peak Type	Peak Codes
1		2.460	7349152	100.00	1032810	bb			Unknown	

b

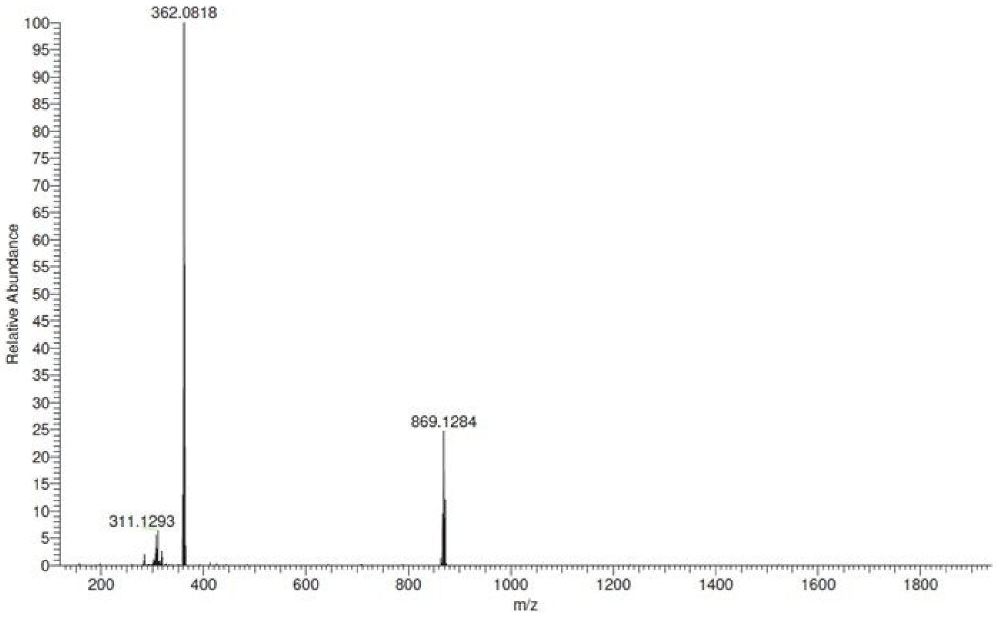

Figure S5. HPLC chromatogram (a) and ESI-MS spectrum (b) of **5**.

a

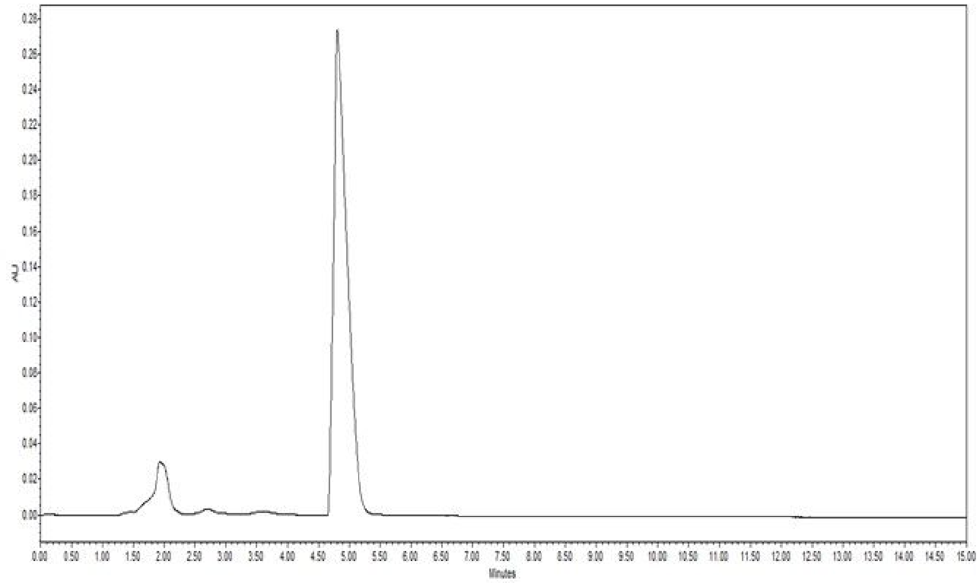

	Name	Retention Time (min)	Area (µV*sec)	% Area	Height (µV)	Int Type	Amount	Units	Peak Type	Peak Codes
1		1.944	229182	5.23	20713	bb			Unknown	I08
2		4.805	4156090	94.77	273724	bb			Unknown	

b

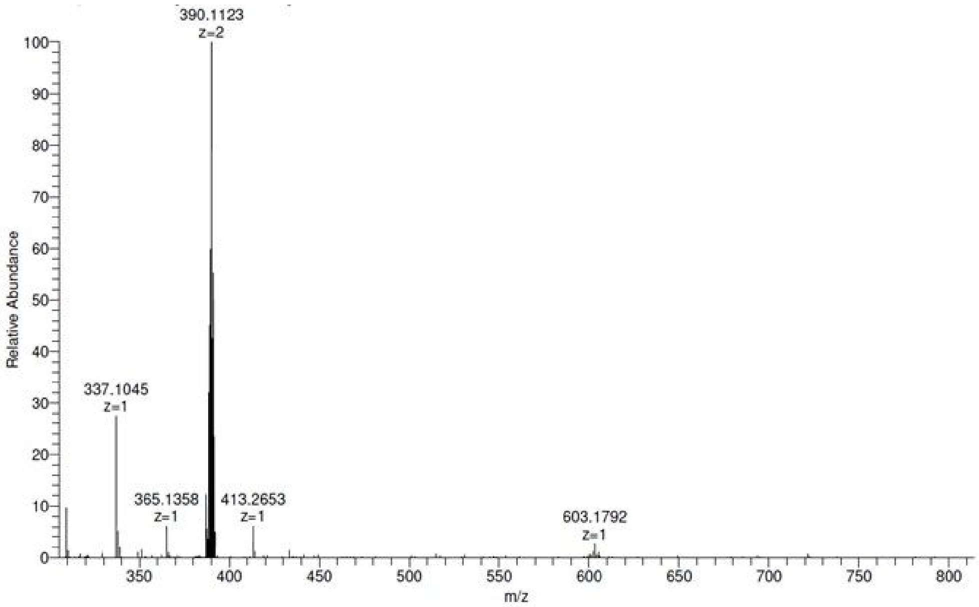

Figure S6. HPLC chromatogram (a) and ESI-MS spectrum (b) of **6**.

a

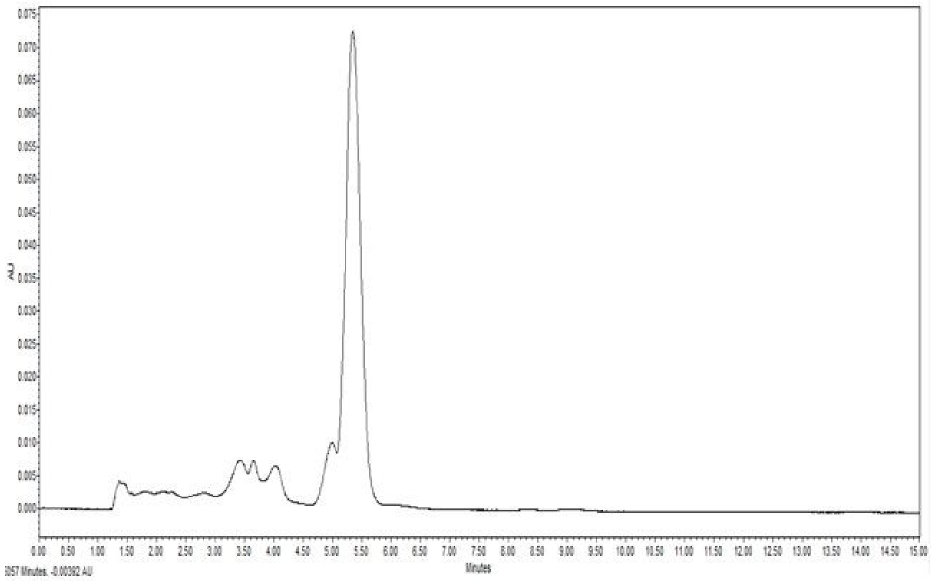

	Name	Retention Time (min)	Area (µV*sec)	% Area	Height (µV)	Int Type	Amount	Units	Peak Type	Peak Codes
1		3.412	45031	3.04	2710	bb			Unknown	
2		4.050	33749	2.28	3139	bb			Unknown	I08
3		5.341	1401336	94.68	71733	bb			Unknown	

b

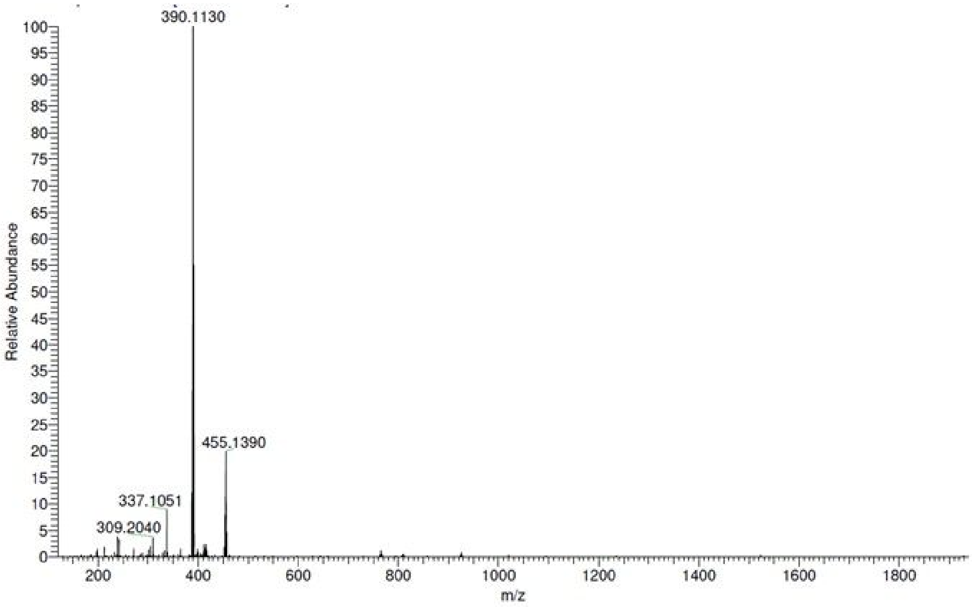

Figure S7. HPLC chromatogram (a) and ESI-MS spectrum (b) of **7**.

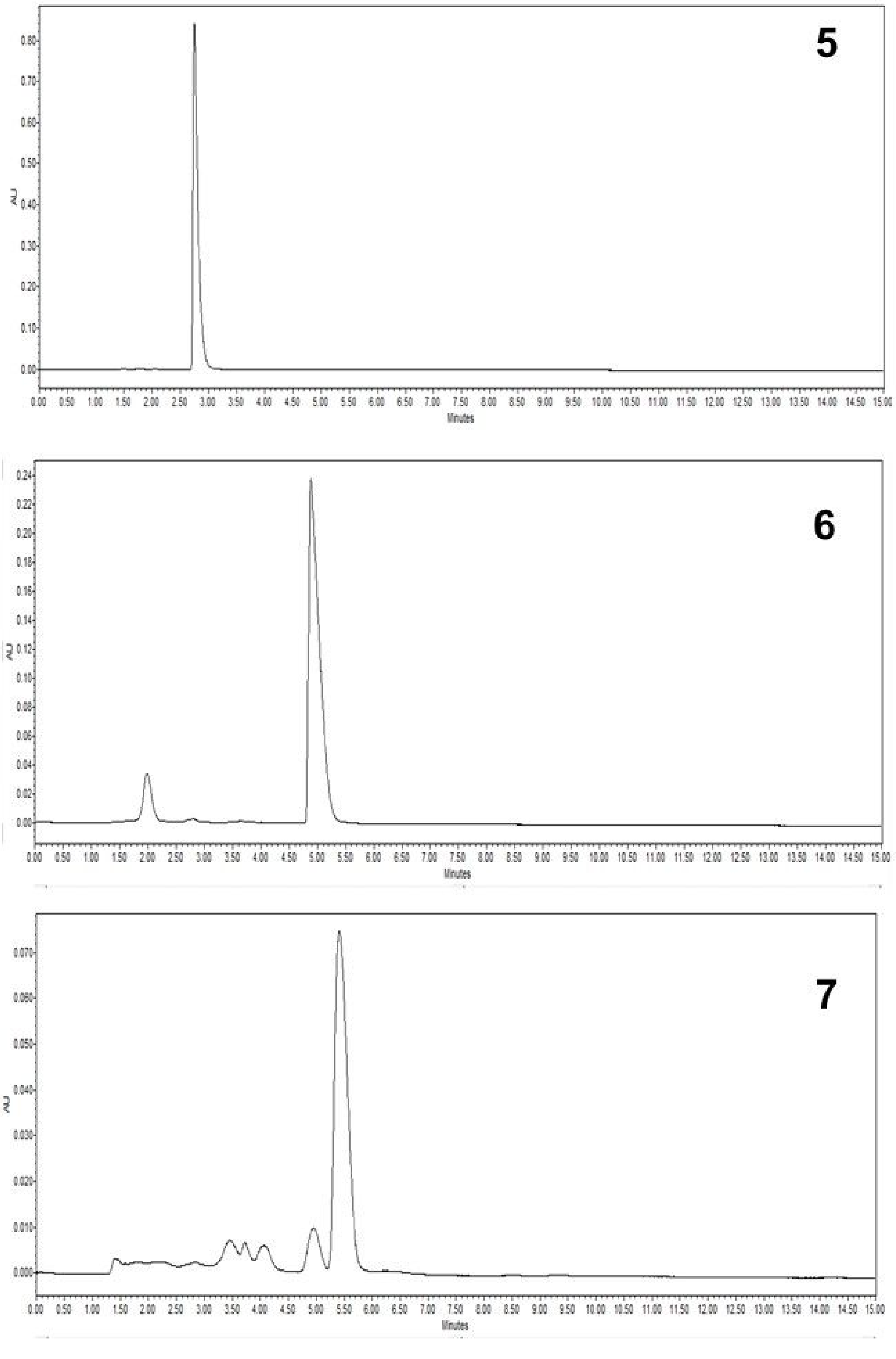

Figure S8. HPLC chromatograms of **5**, **6** and **7** after 14 days in solution.

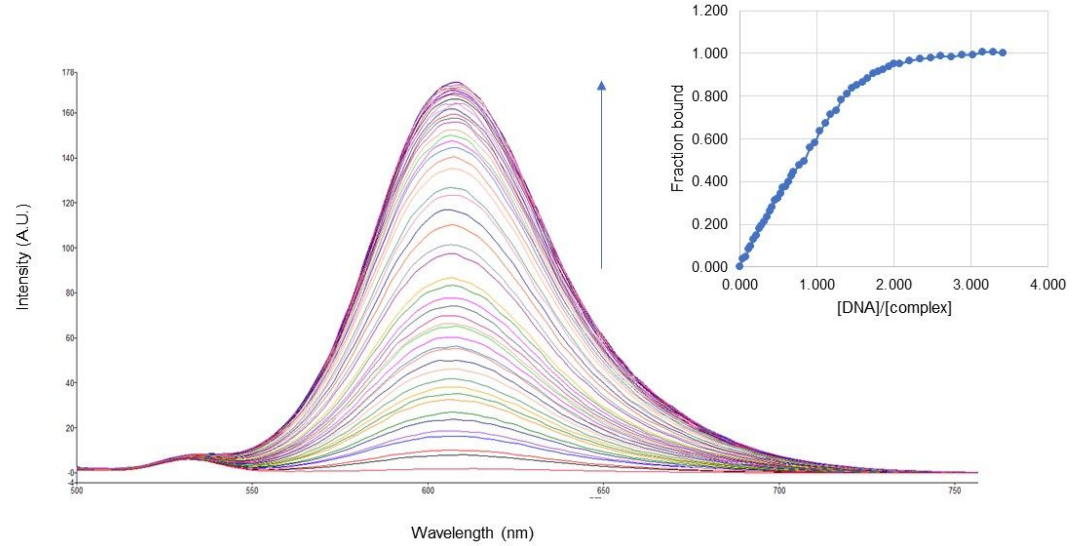

Figure S9. Example of luminescence titration. Emission spectra of **6** (λ_{ex} = 450 nm) with the addition of DNA. Derived binding curve in inset.

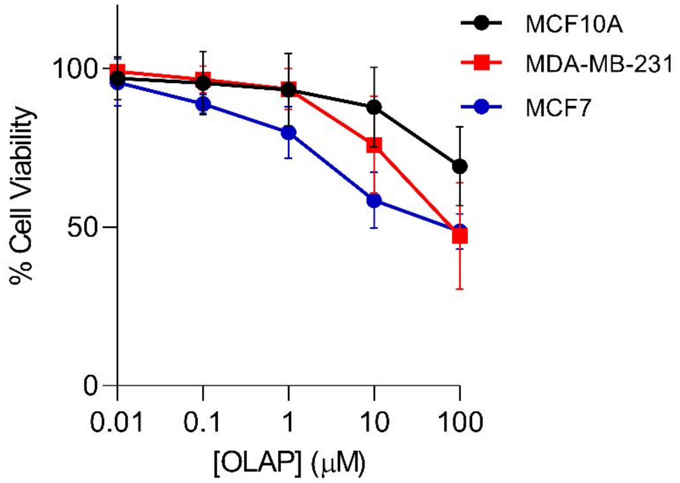

Figure S10. Cell viability of MCF10A, MDA-MB-231 or MCF7 cells upon treatment with Olaparib (OLAP). Cell viability determined after 72 h treatment by MTT assay. Data expressed as mean ± SD of at least two independent experiments.

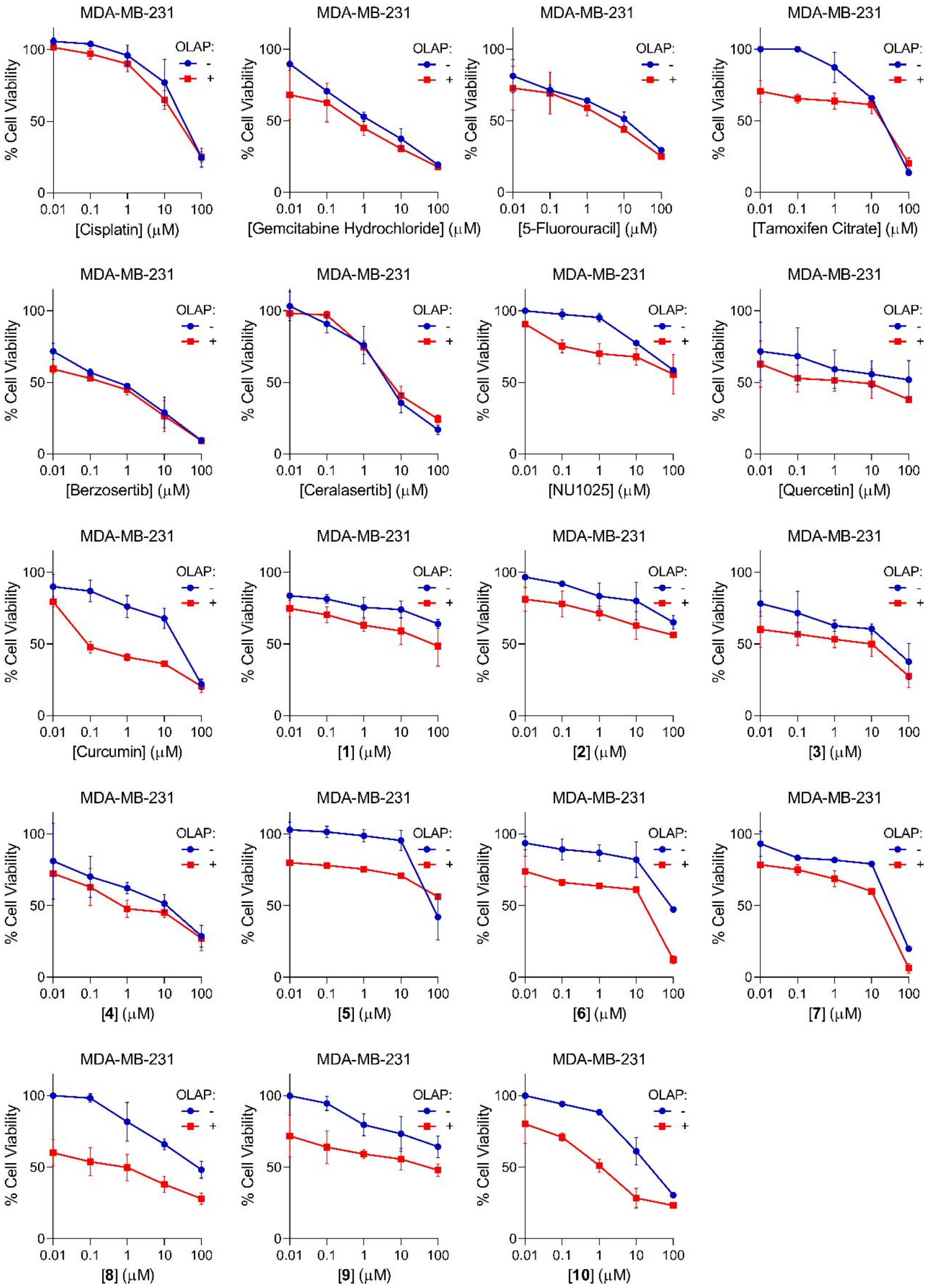

Figure S11. Cell viability of MDA-MB-231 cells treated with concentration gradient of library compound alone or in combination with Olaparib. Cell viability determined after 72 h treatment by MTT assay. Data expressed as mean ± SD of at least two independent experiments.

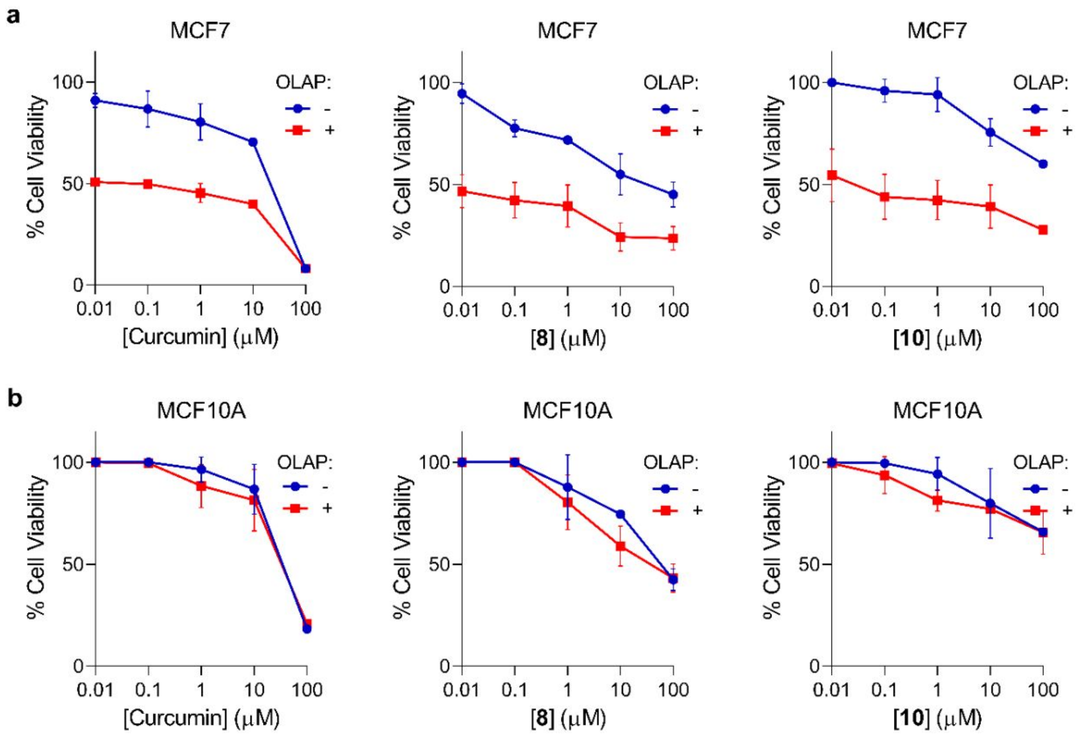

Figure S12. Cell viability of MCF7 breast cancer cells (a) or MCF10A normal human breast cells (b) treated with concentration gradient of Curcumin, **8** or **10** alone or in combination with 10 µM Olaparib. Cell viability determined after 72 h treatment by MTT assay. Data expressed as mean ± SD of at least two independent experiments.

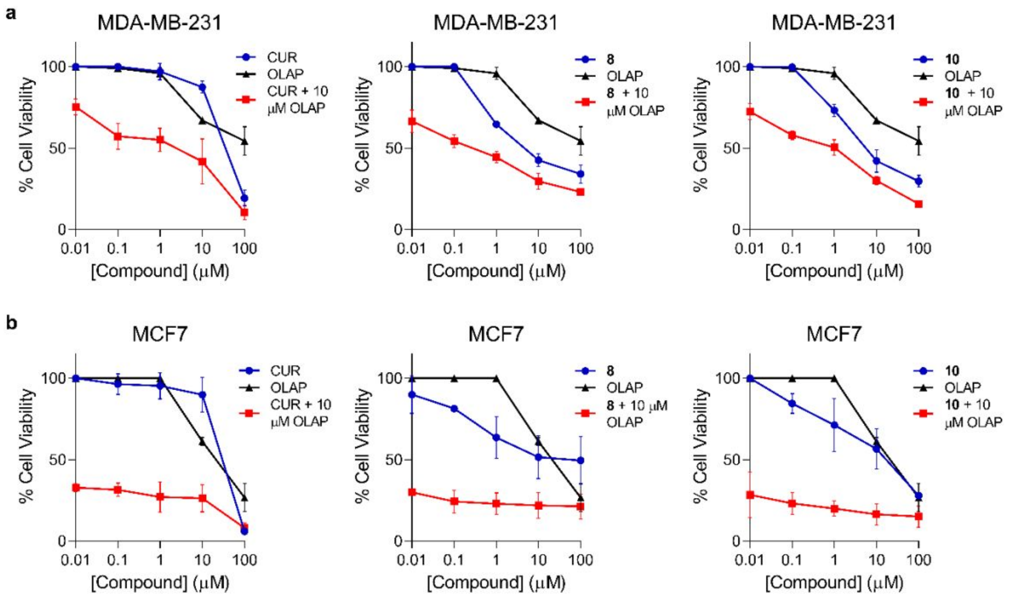

Figure S13. Recovery assay of MDA-MB-231 (a) or MCF7 (b) breast cancer cells treated with concentration gradient of Curcumin, **8** or **10** alone or in combination with Olaparib. Cells were treated for 72 h, solutions were removed, and cells were grown in compound-free medium for a further 48 h. Cell viability was determined by MTT assay. Data expressed as mean ± SD of at least two independent experiments.

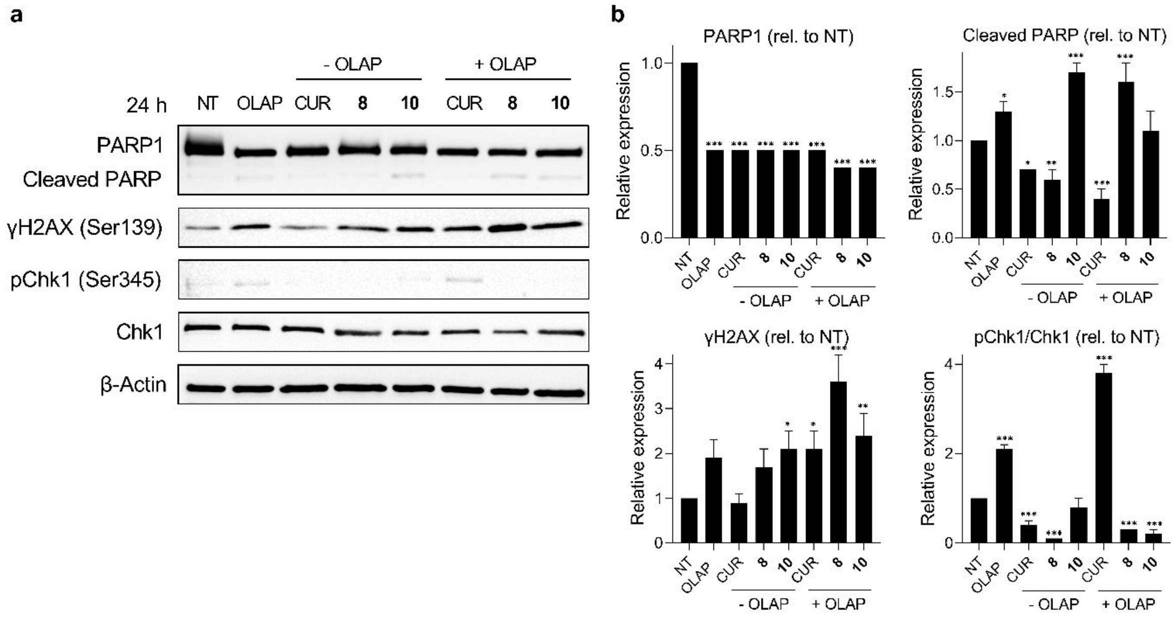

Figure S14. a) Western blot analysis of selected DNA damage response activation in MDA-MB-231 cells treated with CUR, **8** or **10** alone or in combination with OLAP, 24 h treatment. b) Quantification of results in (a) by densitometry. Data expressed as mean ± SD for triplicate experiments. NT = untreated. *P < 0.05, **P < 0.01, ***P < 0.001 compared to (untreated) NT group by ANOVA.

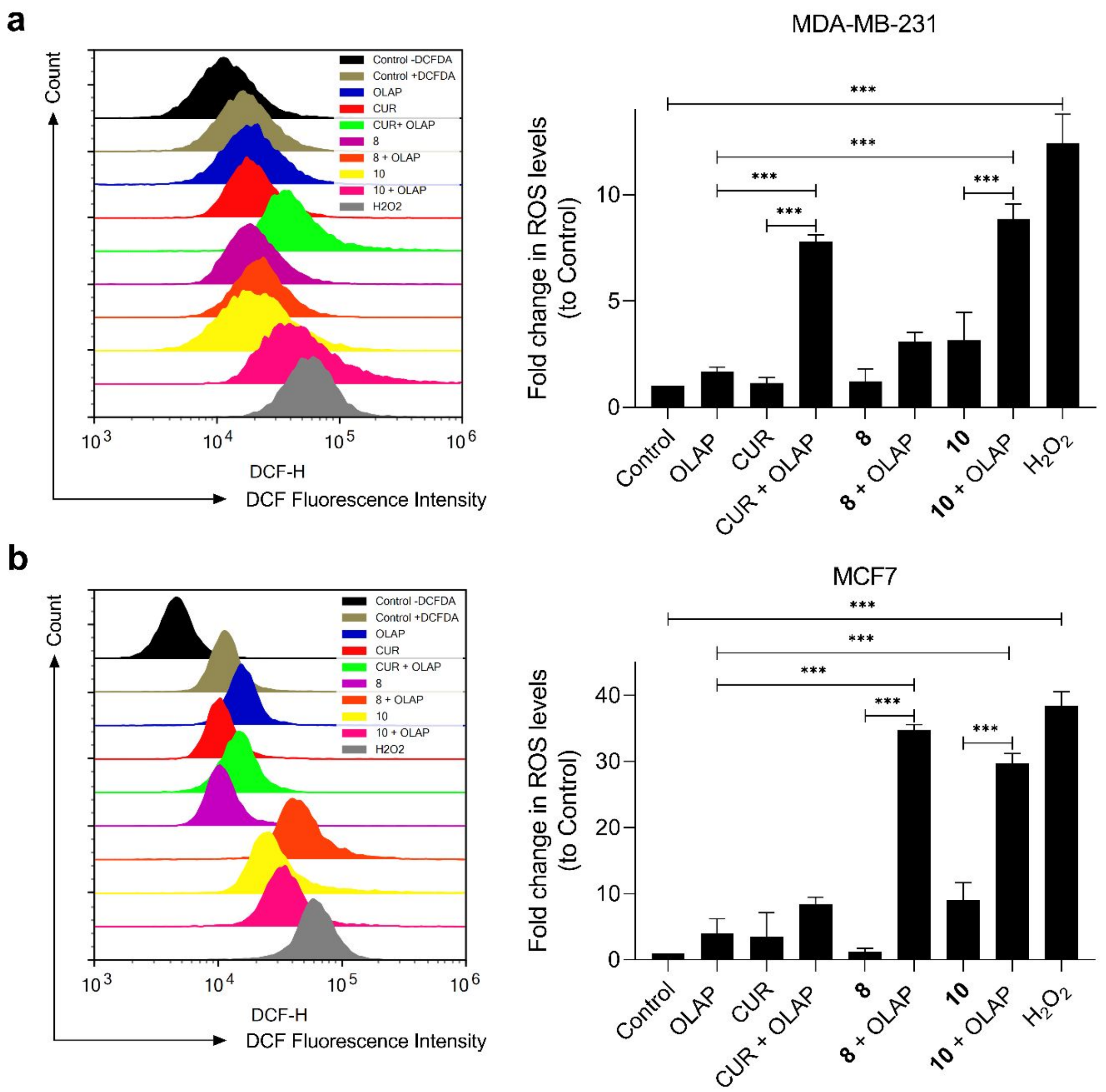

Figure S15. ROS levels of MDA-MB-231 (a) and MCF7 (b) cells treated with stated single-agent (1 µM) alone or in combination with Olaparib (10 µM) for 24 h, as determined by (2',7'-dichlorofluorescein, DCF) staining and flow cytometry. Left, representative histograms, right, quantification of DCF fluorescence intensity in cell populations. Data mean ± SD of two independent experiments. ***$P < 0.001$ by ANOVA.

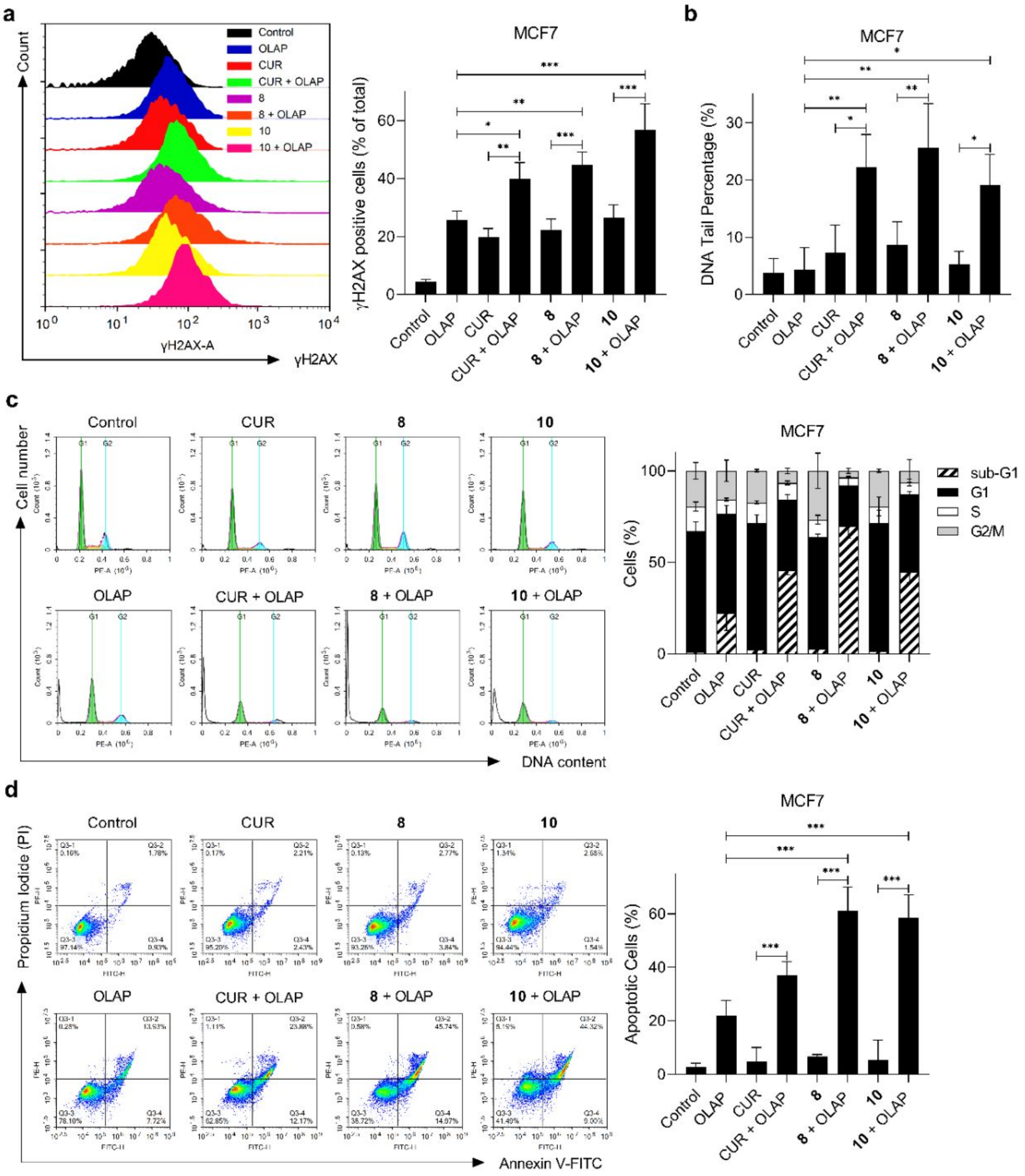

Figure S16. a) γH2AX levels upon treatment with the stated single-agent (1 µM) alone or in combination with Olaparib (10 µM) for 24 h in MCF7 cells, determined by immunofluorescence and flow cytometry analysis. The percentage of γH2AX-positive cells in each population was determined by gating of histograms derived from single-stained cells. Left, representative histograms, right, quantified data. b) Quantification of DNA damage by alkaline comet assay for cells treated as in (a). DNA damage assessed by DNA tail % where at least one hundred nucleoids were analysed per sample. c) Cell-cycle distribution of MFC7 cells treated with stated single-agent (1 µM) alone or in combination with Olaparib (10 µM) for 72 h, as determined by PI staining and flow cytometry. Left, representative histograms, right, quantification of cell-cycle phase. d) Annexin V-FITC assay of MCF7 cells treated as in (c). Left, representative scatterplots showing the percentage of cells in each quadrant. Right, quantification of apoptotic cells (Q3-2 and Q3-4 quadrants) for each treatment condition. Data expressed as mean ± SD of three independent experiments. **P* < 0.05, ***P* < 0.01 and ****P* < 0.001 by ANOVA.

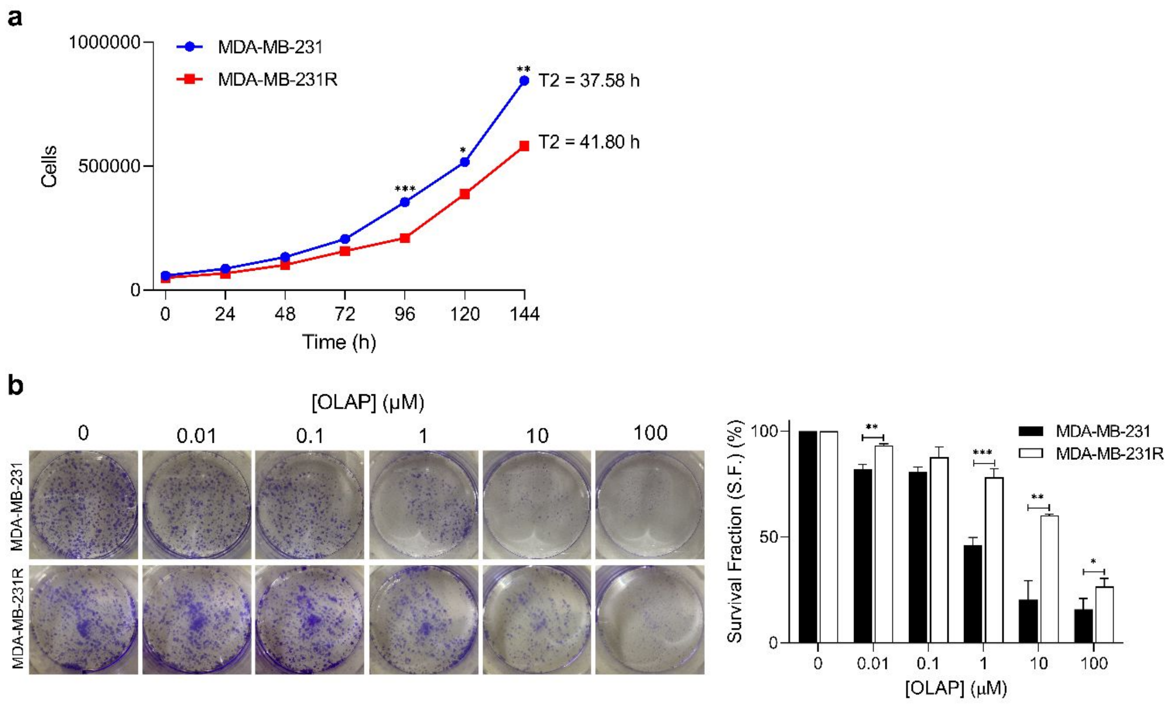

Figure S17. a) Cell doubling time of MDA-MB-231R and native MDA-MB-231 cells. T2 = doubling time. b) Validation of Olaparib-resistant MDA-MB-231 (MDA-MB-231R) by clonogenic survival assay. Representative clonogenic images of MDA-MB-231 and MDA-MB231R cells treated with Olaparib (0, 0.01, 0.1, 1, 10, 100 μM) for eleven days (left) and quantified data (right) were shown. Data expressed as mean ± SD of three independent experiments. **P* < 0.05, ***P* < 0.01 and ****P* < 0.001 by student's *t*-test.

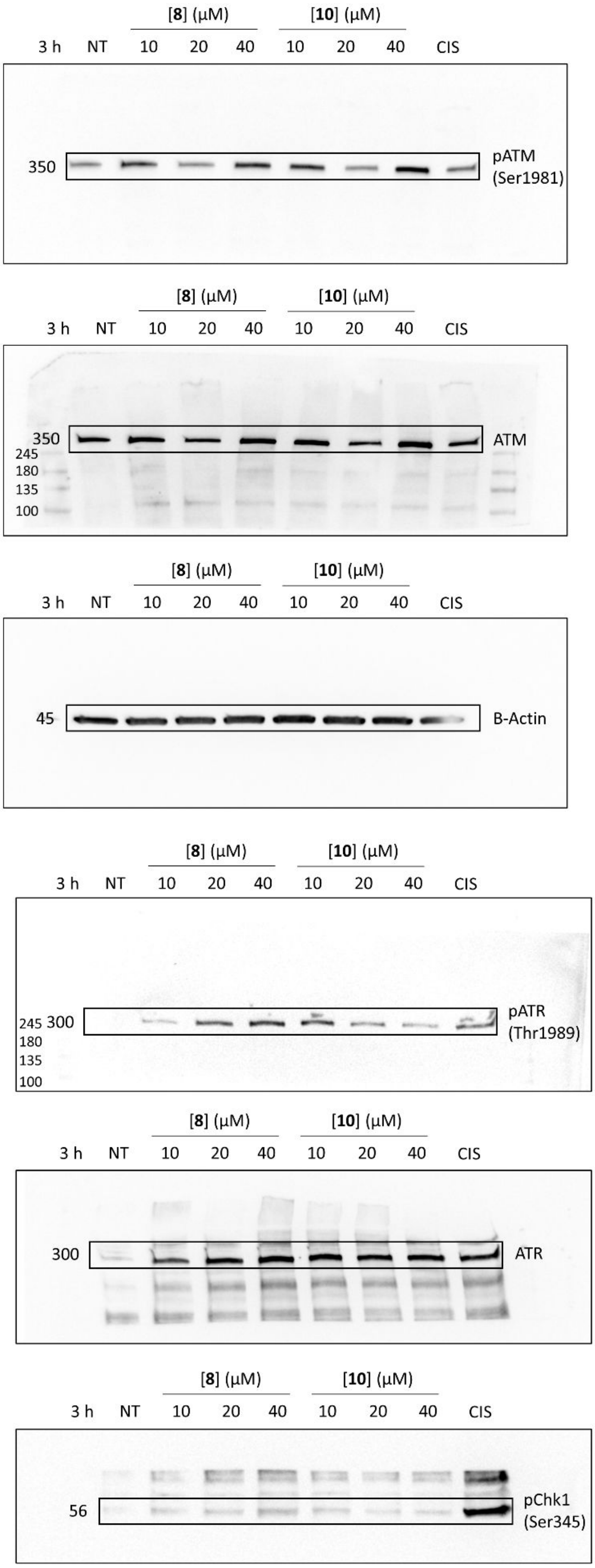
[8] (µM)
[10] (µM)
3 h NT 10 20 40 10 20 40 CIS
350
pATM (Ser1981)
[8] (µM)
[10] (µM)
3 h NT 10 20 40 10 20 40 CIS
350
245
180
135
100
ATM
[8] (µM)
[10] (µM)
3 h NT 10 20 40 10 20 40 CIS
45
B-Actin
[8] (µM)
[10] (µM)
3 h NT 10 20 40 10 20 40 CIS
245 300
180
135
100
pATR (Thr1989)
[8] (µM)
[10] (µM)
3 h NT 10 20 40 10 20 40 CIS
300
ATR
[8] (µM)
[10] (µM)
3 h NT 10 20 40 10 20 40 CIS
56
pChk1 (Ser345)

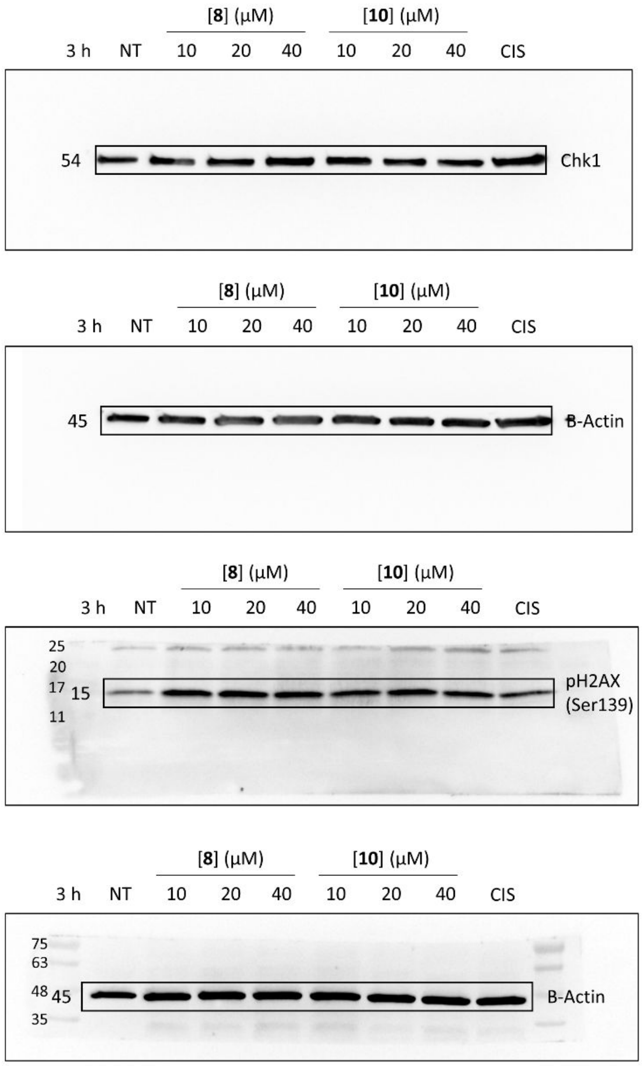

Figure S18. Full blots for Figure 2a in the main text. Molecular weights (kDa) indicated on left hand side.

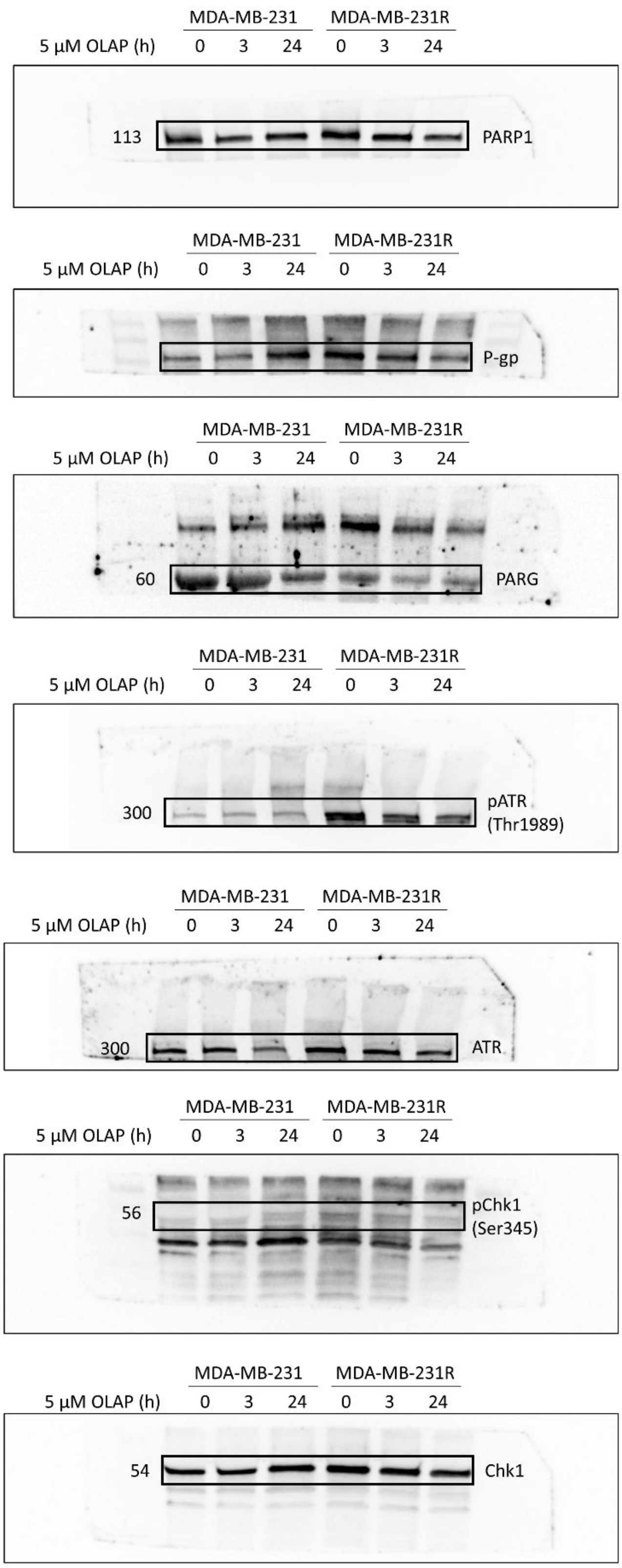
MDA-MB-231
MDA-MB-231R
5 µM OLAP (h)
0
3
24
0
3
24
113
PARP1
MDA-MB-231
MDA-MB-231R
5 µM OLAP (h)
0
3
24
0
3
24
P-gp
MDA-MB-231
MDA-MB-231R
5 µM OLAP (h)
0
3
24
0
3
24
60
PARG
MDA-MB-231
MDA-MB-231R
5 µM OLAP (h)
0
3
24
0
3
24
300
pATR
(Thr1989)
MDA-MB-231
MDA-MB-231R
5 µM OLAP (h)
0
3
24
0
3
24
300
ATR
MDA-MB-231
MDA-MB-231R
5 µM OLAP (h)
0
3
24
0
3
24
56
pChk1
(Ser345)
MDA-MB-231
MDA-MB-231R
5 µM OLAP (h)
0
3
24
0
3
24
54
Chk1

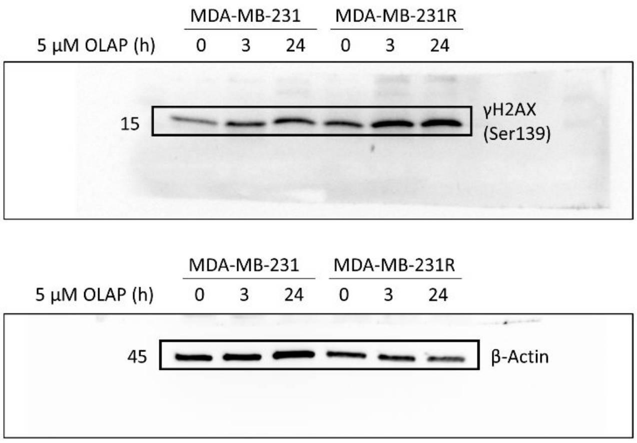

Figure S19. Full blots for Figure 4a in the main text. Molecular weights (kDa) indicated on left hand side.

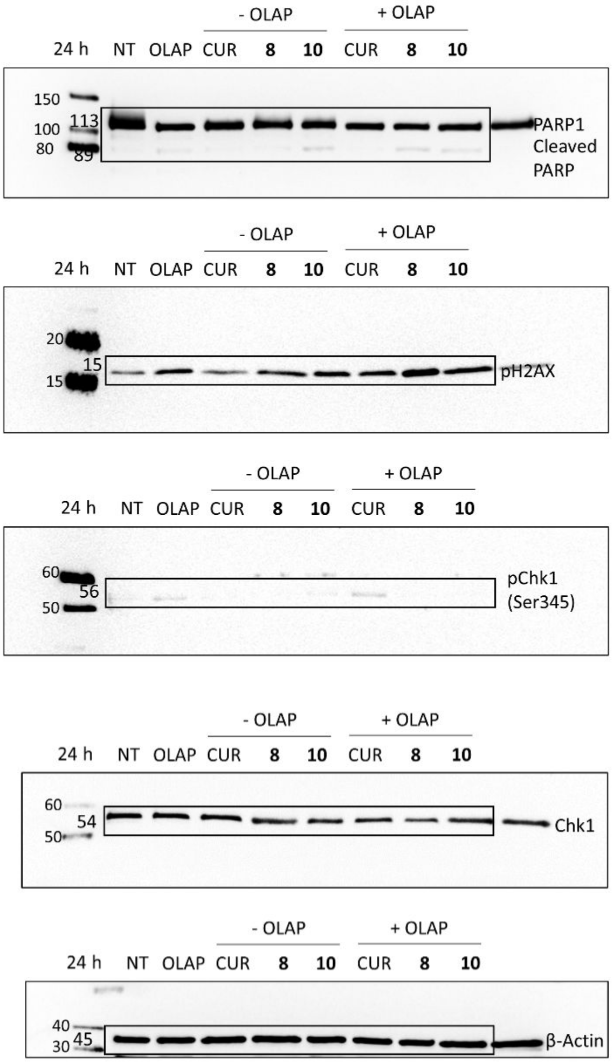

Figure S20. Full blots for Figure S14a in the supplementary figures. Molecular weights (kDa) indicated on left hand side.

References

(1) Wozniak, K.; Kolacinska, A.; Blasinska-Morawiec, M.; Morawiec-Bajda, A.; Morawiec, Z.; Zadrozny, M.; Blasiak, J. The DNA-Damaging Potential of Tamoxifen in Breast Cancer and Normal Cells. *Arch. Toxicol.* **2007**, *81* (7), 519–527.

(2) Srivastava, S.; Somasagara, R. R.; Hegde, M.; Nishana, M.; Tadi, S. K.; Srivastava, M.; Choudhary, B.; Raghavan, S. C. Quercetin, a Natural Flavonoid Interacts with DNA, Arrests Cell Cycle and Causes Tumor Regression by Activating Mitochondrial Pathway of Apoptosis. *Sci. Rep.* **2016**, *6* (1), 24049.

(3) Ting, C.-Y.; Wang, H.-E.; Yu, C.-C.; Liu, H.-C.; Liu, Y.-C.; Chiang, I.-T. Curcumin Triggers DNA Damage and Inhibits Expression of DNA Repair Proteins in Human Lung Cancer Cells. *Anticancer Res.* **2015**, *35* (7), 3867 LP – 3873.

(4) Ji, L.; Zhang, Q.; Liu, J. DNA Structure, Binding Mechanism and Biology Functions of Polypyridyl Complexes in Biomedicine. *Sci. China Ser. B Chem.* **2001**, *44* (3), 246–259.

(5) Liu, J.; Zheng, W.; Shi, S.; Tan, C.; Chen, J.; Zheng, K.; Ji, L. Synthesis, Antitumor Activity and Structure–Activity Relationships of a Series of Ru(II) Complexes. *J. Inorg. Biochem.* **2008**, *102* (2), 193–202.

(6) Ghosh, D.; Ahmad, H.; A. Thomas, J. Kinetically Locked Luminescent Metallomacrocycles as Duplex DNA Binding Substrates. *Chem. Commun.* **2009**, No. 20, 2947–2949.

(7) Ahmad, H.; Ghosh, D.; Thomas, J. A. Using Ancillary Ligands to Tune the DNA Binding Properties of Self-Assembled Luminescent Metallomacrocycles. *Chem. Commun.* **2014**, *50* (29), 3859–3861.
